# Supplementary material for: DSK2-mediated degradation of F-box protein LAO1 and class I TCPs modulates the nitrogen starvation response
Source: EMBO Rep. 2025 May 30;26(14):3614–39. doi: 10.1038/s44319-025-00491-9 (PMC12287301; doi:10.1038/s44319-025-00491-9)
Supplement: Supplementary file 1 — Appendix [file 44319_2025_491_MOESM1_ESM.pdf]

Appendix

Table of content

Appendix Figure S1. *LAO1* expression level in *LAO1* OE1 and OE2 transgenic lines. 1
Appendix Figure S2. Nitrogen starvation destabilized HA-LAO1 protein. 2
Appendix Figure S3. The degradation rate of HA-LAO1 protein in Col, *atg5-1*, *atg7-3*, and *dsk2 cr* backgrounds. 3
Appendix Figure S4. Mutation types induced by CRISPR-Cas 9 at *DSK2* gene locus in *35S: HA-LAO1* background. 4
Appendix Figure S5. Mutation types induced by CRISPR-Cas9. 5
Appendix Figure S6. Phenotype analyses of *TCPs* OE plant after nitrogen starvation.......................6
Appendix Figure S7. Genetic analysis of DSK2 and TCP during nitrogen starvation. 7
Appendix Table S1: Primers used in this study..8

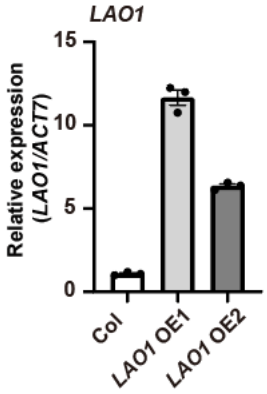

Appendix Figure S1. ***LAO1*** **expression level in** ***LAO1*** **OE1 and OE2 transgenic lines.**

Total RNA was extracted from 7-day-old seedlings for downstream applications. *Actin7* (*ACT7*) was used as internal control. Data were shown in mean ± SEM of 3 technical replicates.

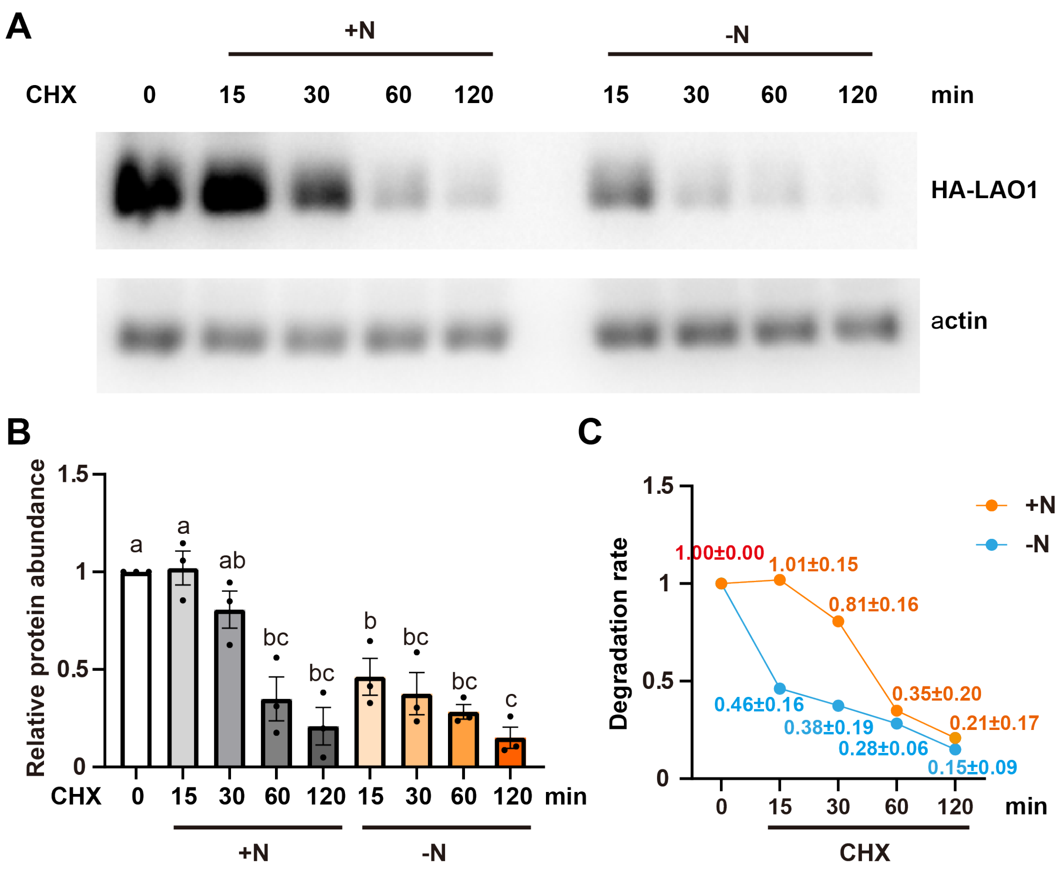

Appendix Figure S2. Nitrogen starvation destabilized HA-LAO1 protein.

Seven-day-old *35S: HA-LAO1* transgenic seedlings grown under continuous white light were used. Seedlings were treated with liquid ½ MS (+N) or ½ MS-N (-N) containing 50 µM Cycloheximide (CHX) for indicated time. Total proteins were extracted and subjected to western blots using anti-HA and anti-actin antibodies. (A) A representative western blot. (B) Relative protein abundance of HA-LAO1 as shown in mean ± SEM of 3 independent experiments. The statistical significance was determined using one-way ANOVA ($p < 0.05$, ANOVA followed by Tukey's post hoc comparison test) analysis. Different letters denoted significant differences. (C) Degradation curve of HA-LAO1 protein. Numbers indicate the relative protein levels of HA-LAO1 (mean ± SD of 3 independent experiments) at each time point compared to time 0.

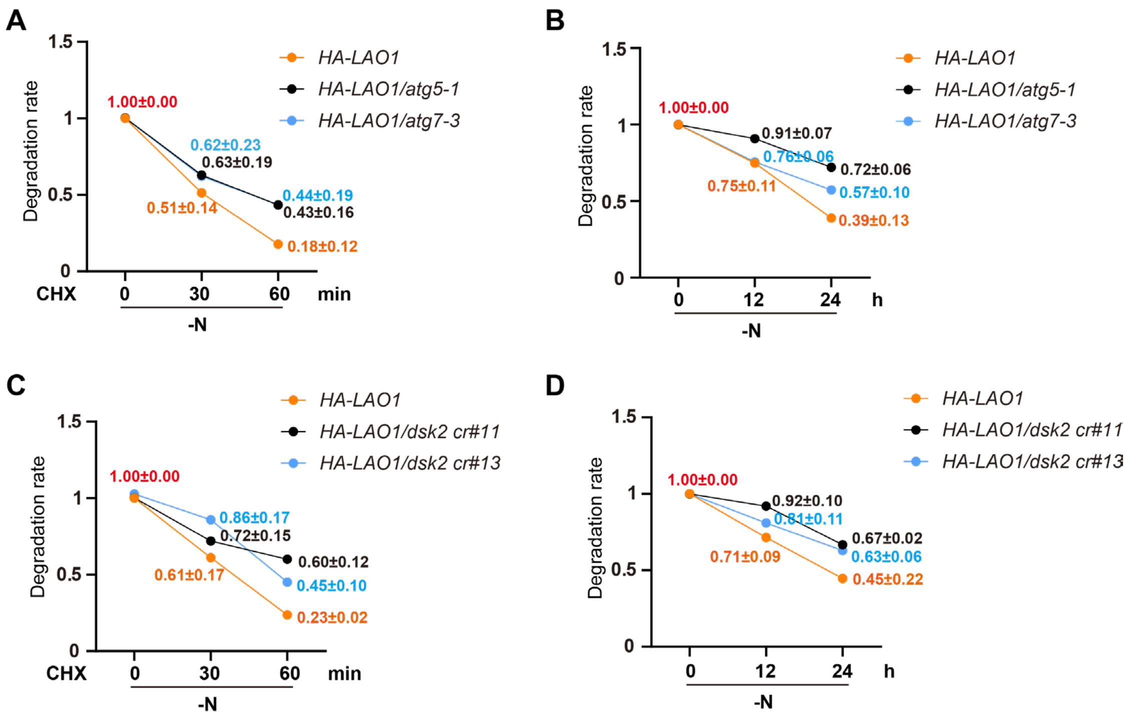

Appendix Figure S3. The degradation rate of HA-LAO1 protein in Col, *atg5-1*, *atg7-3*, and *dsk2 cr* backgrounds.

(A) Degradation curve of HA-LAO1 protein for experiments shown in Figure 3B.
(B) Degradation curve of HA-LAO1 protein for experiments shown in Figure 3C.
(C) Degradation curve of HA-LAO1 protein for experiments shown in Figure 4F.
(D) Degradation curve of HA-LAO1 protein for experiments shown in Figure 4G.

Numbers indicate the relative protein levels of HA-LAO1 (mean ± SD of 3 independent experiments) at each time point compared to time 0.

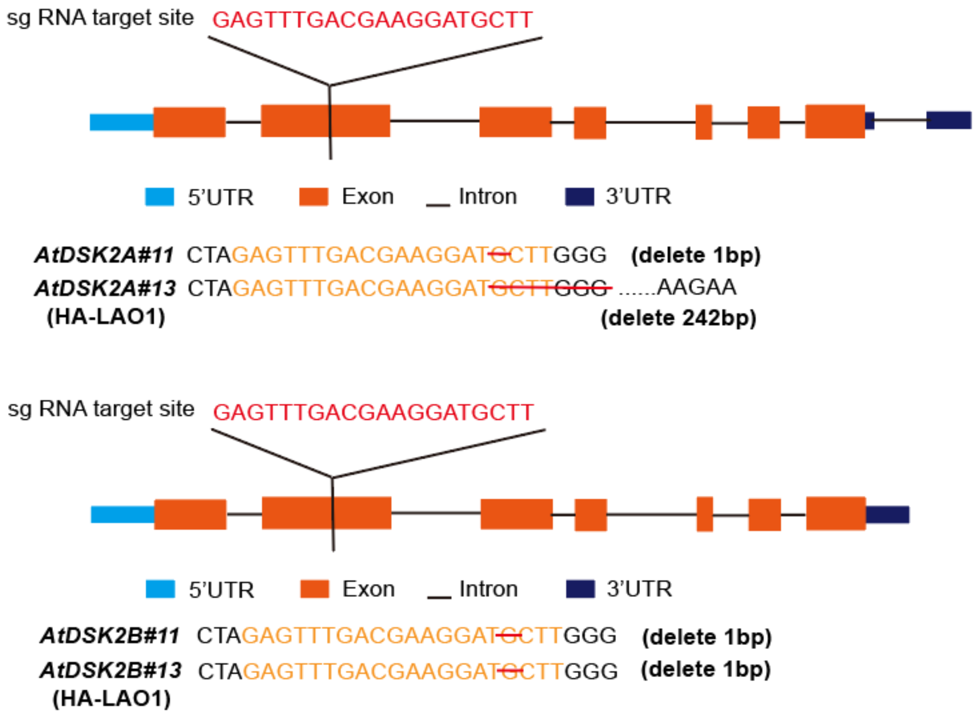

Appendix Figure S4. Mutation types induced by CRISPR-Cas9 at *DSK2* gene locus in *35S: HA-LAO1* background.

The gene structure of *DSK2* and the position of sgRNA were shown. Mutations were confirmed by PCR-sequencing of targeted locus.

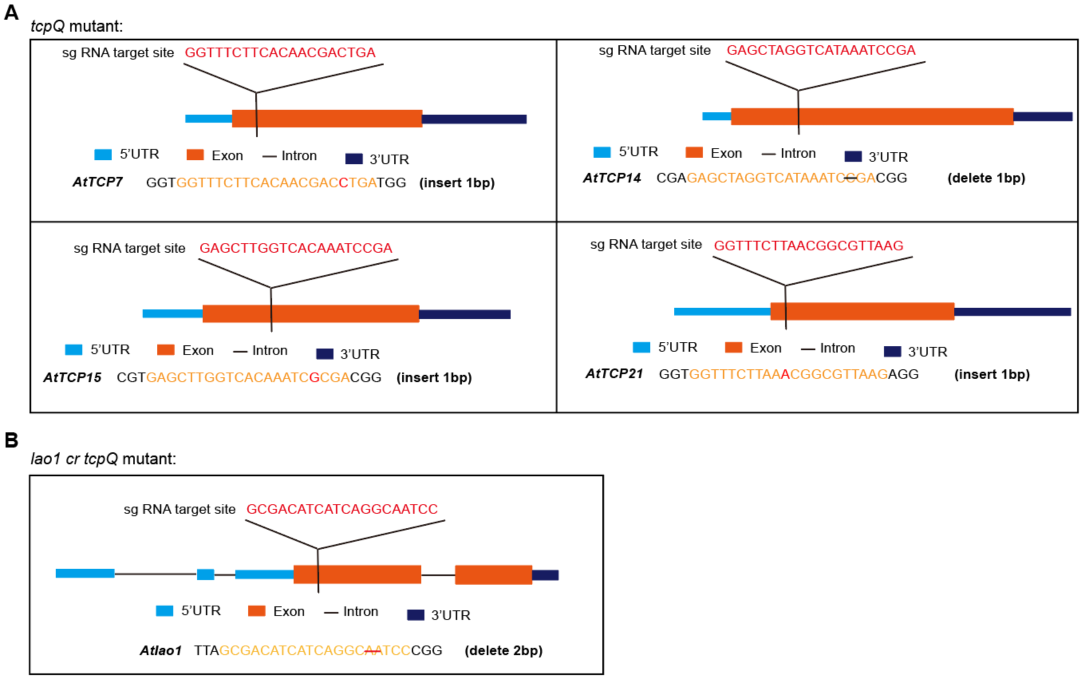

Appendix Figure S5. Mutation types induced by CRISPR-Cas9.

(A) Mutation types induced by CRISPR-Cas 9 at *TCP7, TCP14, TCP15* and *TCP21* gene locus in *tcpQ* mutant. The gene structure of *TCP7, TCP14, TCP15* and *TCP21* and the positions of sgRNA were shown, respectively.
(B) Mutation types induced by CRISPR-Cas 9 at *LAO1* gene locus in *lao1 cr tcpQ* mutant. All the mutations were confirmed by PCR-sequencing of targeted locus.

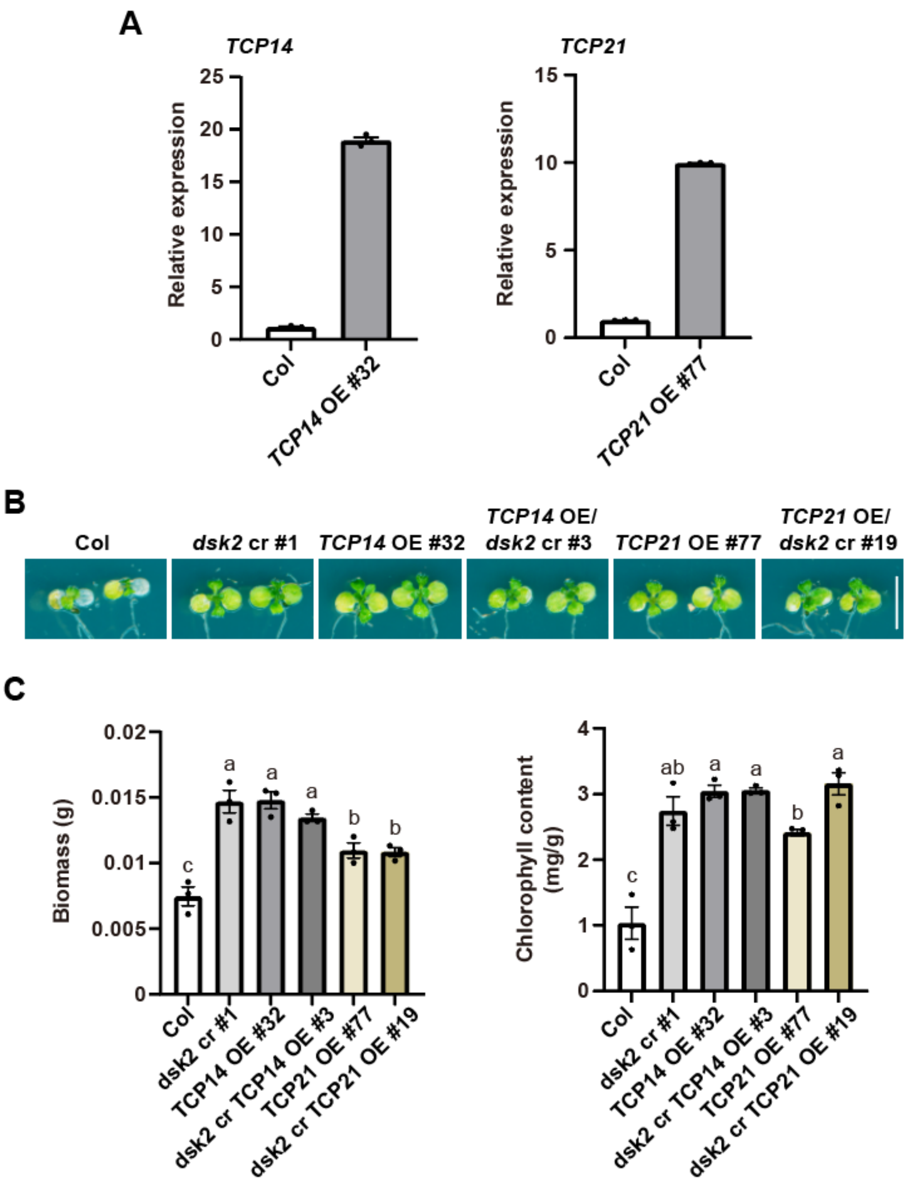

Appendix Figure S6. Phenotype analyses of *TCPs* OE plant after nitrogen starvation.

(A) RT-qPCR analyses of *TCP14* and *TCP21* transcript levels in *TCP14* OE #32 and *TCP21* OE #77 transgenic lines. Total RNA was extracted from 7-day-old seedlings for downstream applications. *Actin7* (*ACT7*) was used as internal control. Data were shown in mean ± SEM of 3 technical replicates. (B) Seedling phenotypes of indicated genotype after nitrogen starvation for six days followed by four days of recovery. Scale bar, 1 cm. (C)The biomass and chlorophyll content of each genotype shown in mean ± SEM of 3 independent experiments. Each independent experiment contained at least 15 seedlings. All statistical significance was determined using one-way ANOVA analysis ($p < 0.05$, ANOVA followed by Tukey's post hoc comparison test). Different letters denoted significant differences.

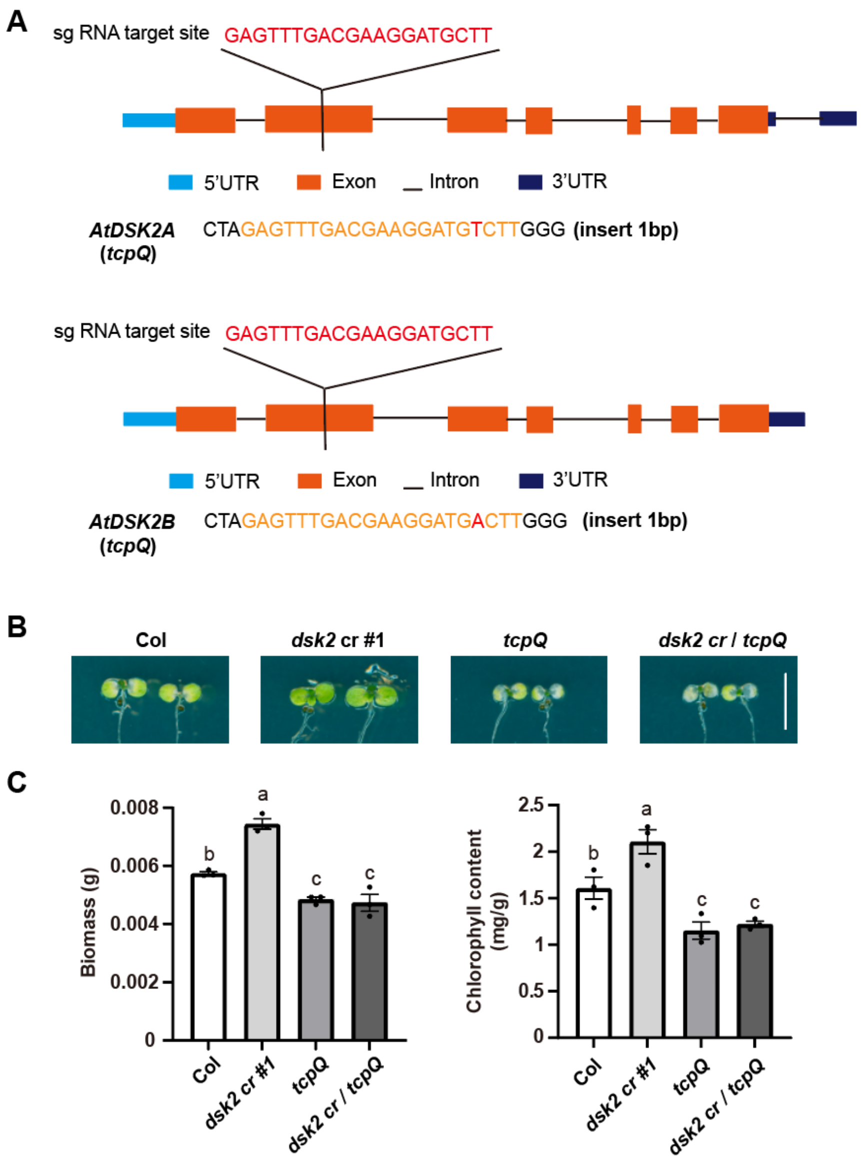

Appendix Figure S7. Genetic analysis of DSK2 and TCP during nitrogen starvation.

(A) Mutation types induced by CRISPR-Cas 9 at *DSK2* gene locus in *dsk2 cr tcpQ* mutant. All the mutations were confirmed by PCR-sequencing of targeted locus. (B, C) Four-day-old seedlings were treated with nitrogen starvation for five days followed by two days of recovery. (B) Representative images. Scale bars: 0.5 cm. (C) The biomass and chlorophyll content of each genotype shown in mean ± SEM of 3 independent experiments. Each independent experiment contained at least 15 seedlings. Statistical significance was determined using one-way ANOVA ($p < 0.05$, ANOVA followed by Tukey's post hoc comparison test) analysis. Different letters denoted significant differences.

Appendix Table S1: Primers used in this study

Name	Primer sequence (5’-3’)
**For Y2H screen**	
PGBKT7-LAO1 CT-F	ATGGCCATGGAGGCCGAATTC GCTGGTATGCTTGAGGAG
PGBKT7-LAO1 CT-R	TCGACGGATCCCCGGGAATTC TCAGGCTGTGATTGTGAC
**For Y2H assay**	
PGADT7-DSK2NT-F	GCCATGGAGGCCAGTGAATTC ATGGGTGGAGAGGGAGATTC
PGADT7-DSK2NT-R	ATGCCCACCCGGGTGGAATTC CATTTCACGCATAAGCTCTG
PGADT7-DSK2CT-F	GCCATGGAGGCCAGTGAATTC ATGCGGAATACTGATAGGGC
PGADT7-DSK2CT-R	ATGCCCACCCGGGTGGAATTC CTACTGTCCGATACTCCCCA
PGBKT7-DSK2-F	ATGGCCATGGAGGCCGAATTC ATGGGTGGAGAGGGAGATTC
PGBKT7-DSK2-R	TCGACGGATCCCCGGGAATTC CTACTGTCCGATACTCCCCA
PGADT7-TCP7-F	GCCATGGAGGCCAGTGAATTC ATGTCTATTAACAACAACAAC
PGADT7-TCP7-R	ATGCCCACCCGGGTGGAATTC TTAACGTGGATCTTCCTCTC
PGADT7-TCP14-F	GCCATGGAGGCCAGTGAATTC ATGCAAAAGCCAACATCAAG
PGADT7-TCP14-R	ATGCCCACCCGGGTGGAATTC CTAATCTTGCTGATCCTCCT
PGADT7-TCP15-F	GCCATGGAGGCCAGTGAATTC ATGGATCCGGATCCGGATCATAAC
PGADT7-TCP15-R	ATGCCCACCCGGGTGGAATTC CTAGGAATGATGACTGGTGC
PGADT7-TCP21-F	GCCATGGAGGCCAGTGAATTC ATGGCCGACAACGACGGAGC
PGADT7-TCP21-R	ATGCCCACCCGGGTGGAATTC TCAACGTGGTTCGTGGTCGT
**For GUS histochemical staining**	
proLAO1(3K)-GUS-F	TTGGGCCCGGCGCGCCG GCTACCACATAACTTGTC
proLAO1(3K)-GUS-R	TTGGCTGCAGGTCGACG CCCGTCACGTATAGTGAT

proLAO1(2K)-GUS-F	TTGGGCCCGGCGCGCCG CACCATCATCTGATCTGATG
**For constructing overexpression vectors**	
LAO1-mCherry-F	CTGTACAAGTCTAGAGGATCC ATGCAAGCTTTTGCTTTAAGT
LAO1-mCherry-R	GGGAAATTCGAGCTCGGTACC TCAGGCTGTGATTGTGACACT
TCP14-GFP-F	CTGTACAAGTCTAGAGGATCC ATGCAAAAGCCAACATCAAG
TCP14-GFP-R	GAGCTCGGTACCCGGG CTAATCTTGCTGATCCTCCT
TCP15-4MYC/GFP-F	GGCGACCTCACCATGTCTAGA GATCCGGATCCGGATCATAA
TCP15-4MYC/GFP-R	GGTACCCGGGGATCCTCT GGAATGATGACTGGTGCTTCC
TCP21-GFP-F	CTGTACAAGTCTAGAGGATCC ATGGCCGACAACGACGGAGC
TCP21-GFP-R	GAGCTCGGTACCCGGG TCAACGTGGTTCGTGGTCGT
DSK2-GFP-F	CTGTACAAGTCTAGAGGATCC ATGGGTGGAGAGGGAGATTC
DSK2-GFP-R	GAGCTCGGTACCCGGG CTACTGTCCGATACTCCCCA
**For Dual-luciferase**	
pLAO1(2K)-LUC-F	GACGGTATCGATAAGCTTGA CACCATCATCTGATCTGATG
pLAO1(3K)-LUC-F	GACGGTATCGATAAGCTTGA GCTACCACATAACTTGTC
pLAO1(2K/3K)-LUC-R	GGAATTCGATATCAAGCTTA CCCGTCACGTATAGTGAT
pLAO1(2K/3K)-LAO1-LUC-F	TGCAGCCCGGGGGATCCA ATGCAAGCTTTTGCTTTAAGT
pLAO1(2K/3K)-LAO1-LUC-R	CTCTAGAACTAGTGGATCCCC GGCTGTGATTGTGACACTGCA
UBQ-LAO1-LUC-F	TGCAGCCCGGGGGATCCA ATGCAAGCTTTTGTAAGTGGG
UBQ-LAO1-LUC-R	CTCTAGAACTAGTGGATCCCC GGCTGTGATTGTTACGCTAC
UBQ-TCP7-LUC-F	TGCAGCCCGGGGGATCCA ATGTCTATTAACAACAACAAC
UBQ-TCP7-LUC-R	CTCTAGAACTAGTGGATCCCC ACGTGGATCTTCCTCTCTTC
UBQ-TCP14-LUC-F	TGCAGCCCGGGGGATCCA

	ATGCAAAAGCCAACATCAAG
UBQ-TCP14-LUC-R	CTCTAGAACTAGTGGATCCCC
	ATCTTGCTGATCCTCCTCAT
UBQ-TCP15-LUC-F	TGCAGCCCGGGGGATCCA
	ATGGATCCGGATCCGGATCA
UBQ-TCP15-LUC-R	CTCTAGAACTAGTGGATCCCC
	GGAATGATGACTGGTGCTTC
UBQ-TCP21-LUC-F	TGCAGCCCGGGGGATCCA
	ATGGCCGACAACGACGGA
UBQ-TCP21-LUC-R	CTCTAGAACTAGTGGATCCCC
	ACGTGGTTCGTGGTCGTCTT
**For protein purification**	
DSK2-His-F	GCCATGGCTGATATCGGATCC
	ATGGGTGGAGAGGGAGATTC
DSK2-His-R	TGCGGCCGCAAGCTTGTCGA
	CTACTGTCCGATACTCCCCA
TCP7-His-F	GCTGATATCGGATCCGAATTC
	ATGTCTATTAACAACAACAAC
TCP7-His-R	TTGTCGACGGAGCTCGAA
	TTAACGTGGATCTTCCTCTCT
TCP14-His-F	GCCATGGAGGCCAGTGAATTC
	ATGCAAAAGCCAACATCAAG
TCP14-His-R	ATGCCCACCCGGGTGGAATTC
	CTAATCTTGCTGATCCTCCT
TCP15-His-F	GCCATGGCTGATATCGGATCC
	ATGGGTGGAGAGGGAGATTC
TCP15-His-R	TTGTCGACGGAGCTCGAA
	CTAGGAATGATGACTGGTGCT
TCP21-His-R	GCTGATATCGGATCCGAATTC
	ATGGCCGACAACGACGGAGCA
TCP21-His-F	TTGTCGACGGAGCTCGAA
	TCAACGTGGTTCGTGGTCGT
DSK2 CT-GST-F	GCGTGGATCCCCGGAATTC
	ATGCGGAATACTGATAGGGC
DSK2 CT-GST-F	CTCGAGTCGACCCGGGAA
	CTACTGTCCGATACTCCCCA
**For qPCR assay**	
ACT7-qPCR-F	TCGTGGTGGTGAGTTTGTTAC
ACT7-qPCR-R	CAGCATCATCACAAGCATCC
TCP14-qPCR-F	GCAAAAGTCACAACCGCAGA
TCP14-qPCR-R	GCTTTTTAGCTGCCACCACC
TCP15-qPCR-F	GTTTACAACACGAGCGGTGG
TCP15-qPCR-R	TGATCTCCACCTCCTCCTCC
TCP21-qPCR-F	TCGTTAATGGGTGGTGGTGG

TCP21-qPCR-R	CGGACGAAGAGTGTAGCTGG
LAO1-qPCR-F	CGCTGGTATGCTTGAGGAGT
LAO1-qPCR-R	CTGTTTTTGCAGGACCAGGC
DSK2-qPCR-F	AAGCCAAACCACTGCACCTA
DSK2-qPCR-R	AGCATTTCCACCACCCAGAG
**For DSK2 RNAi**	
DSK2B-1F-F	CATTTACAATTACCATGG GGAATCTTTGTTCCCTGGGCTTGGAT
DSK2B-1F-R	TCGATTGGGCGCGCCCCA CATTTGTCCTTCCAGGGGCAGTTG
DSK2B-2F-F	CATTTACAATTACCATGG GAGGCCTTGGTGGACTCGGTATGCTTG
DSK2B-2F-R	TCGATTGGGCGCGCCCCA TACTGTCCGATACTCCCCAAGAGTCG
DSK2B-1R-F	TTGCAGGTATTTGGATCC CATTTGTCCTTCCAGGGGCAG
DSK2B-1R-R	CTTAATTAACTCTCTAGA GGAATCTTTGTTCCCTGGGCT
DSK2B-2R-F	CTTAATTAACTCTCTAGA GAGGCCTTGGTGGACTCGGTATGCTTG
DSK2B-2R-R	TTGCAGGTATTTGGATCC TACTGTCCGATACTCCCCAAGAGT
**For identification of T-DNA insertion mutants**	
atg5-1-LP	ATTTGCTATTTGTTTGGCACG
atg5-1-RP	TACCGTTCATGACAGAGGTCC
atg5-1-LB	GCCTTTTCAGAAATGGATAAATAGCCTTG CTTCC
Atg7-3-LP	CAGCGTGATCTGTGAGAACTG
Atg7-3-RP	TTCTTGGAGCTGGTACATTGG
lao1-LP	GCATACGCAAGAACCAGAGAG
lao1-RP	TGATGATGTCGCTAAGCAGTG
lao1-LB	TGATCCATGTAGATTTCCCGGACATGAAG
**For constructing CRISPR Cas9 editing vectors**	
LAO1-CR-F	GATT GCGACATCATCAGGCAATCC
LAO1-CR-R	AAAC GGATTGCCTGATGATGTCGC
DSK2-CR-F	GATT GAGTTTGACGAAGGATGCTT
DSK2-CR-R	AAAC AAGCATCCTTCGTCAAACTC
TCP7-CR-F	GATT GGTTTCTTCACAACGACTGA
TCP7-CR-R	AAAC TCAGTCGTTGTGAAGAAACC
TCP14-CR-F	GATT GAGCTAGGTCATAAATCCGA
TCP14-CR-R	AAAC TCGGATTTATGACCTAGCTC
TCP15-CR-F	GATT GAGCTTGGTCACAAATCCGA

TCP15-CR-R	AAAC TCGGATTTGTGACCAAGCTC
TCP21-CR-F	GATT GGTTTCTTAACGGCGTTAAG
TCP21-CR-R	AAAC CTTAACGCCGTTAAGAAACC
**For CRISPR genotyping**	
LAO1-CR-genotyping-F	ACTGCAAATGCAAACCCACC
LAO1-CR-genotyping-R	TCTCTCTGTTCCCGTTTTCTCTG
DSK2A-CR-genotyping-F	GCTCTTGTTCAGCGTTGTCC
DSK2A-CR-genotyping-R	AAGAAGAGGCTTACAGGCGAC
DSK2B-CR-genotyping-F	ACTTGTAACAAACTGATTCTTCAGC
DSK2B-CR-genotyping-R	TATCAGCAGCAGGGTTTCGAG
TCP7-CR-genotyping-F	TCTGCAATCGAGAACCCACA
TCP7-CR-genotyping-R	ATCAAAACGGATCAATGGCGG
TCP14-CR-genotyping-F	TGAGCACGATGCTTCTCGTT
TCP14-CR-genotyping-R	GCAAAGGGGCCAATGTTGTG
TCP15-CR-genotyping-F	CTCGGGTTGTGTCCGAATCT
TCP15-CR-genotyping-R	GTTATCGGGGAGGATCGGGA
TCP21-CR-genotyping-F	TGGCTCTGGAGCTTTAGTTTGT
TCP21-CR-genotyping-R	GGGAGGTTATGTGCCCAGAG
**For bimolecular fluorescence complementation**	
LAO1-YN-F	TACAAAAAAGCAGGCTTC GCTGGTATGCTTGAGGAGTG
LAO1-YN-R	CAAGAAAGCTGGGTGAGC GGCTGTGATTGTGACACTGC
TCP14-YC-F	TACAAAAAAGCAGGCTTC ATGCAAAAGCCAACATCAAG
TCP14-YC-R	CAAGAAAGCTGGGTGAGC ATCTTGCTGATCCTCCTCAT
TCP21-YC-F	TACAAAAAAGCAGGCTTC ATGGCCGACAACGACGGAGC
TCP21-YC-R	CAAGAAAGCTGGGTGAGC ACGTGGTTCGTGGTCGTCTT
