# Supplementary figures and images for: DSK2-mediated degradation of F-box protein LAO1 and class I TCPs modulates the nitrogen starvation response
Source: EMBO Rep. 2025 May 30;26(14):3614–39. doi: 10.1038/s44319-025-00491-9 (PMC12287301; doi:10.1038/s44319-025-00491-9)

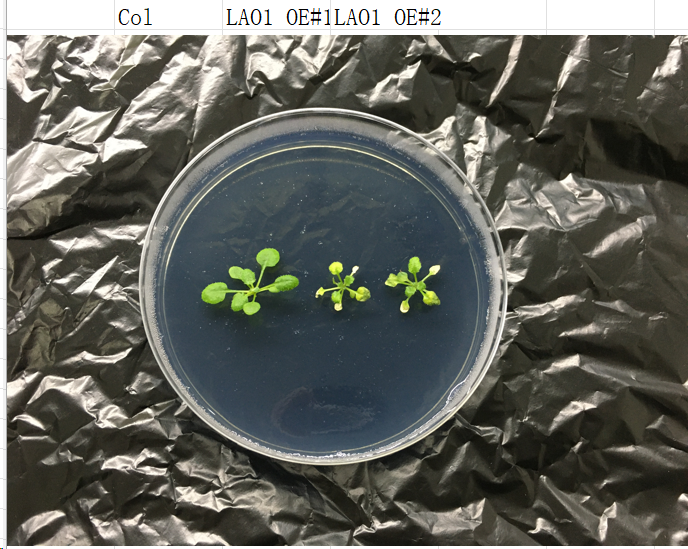

Supplement: Supplementary file 4 — Source data Fig. 1 [file 44319_2025_491_MOESM4_ESM.zip › Figure 1/Figure 1A/Figure 1A.png]

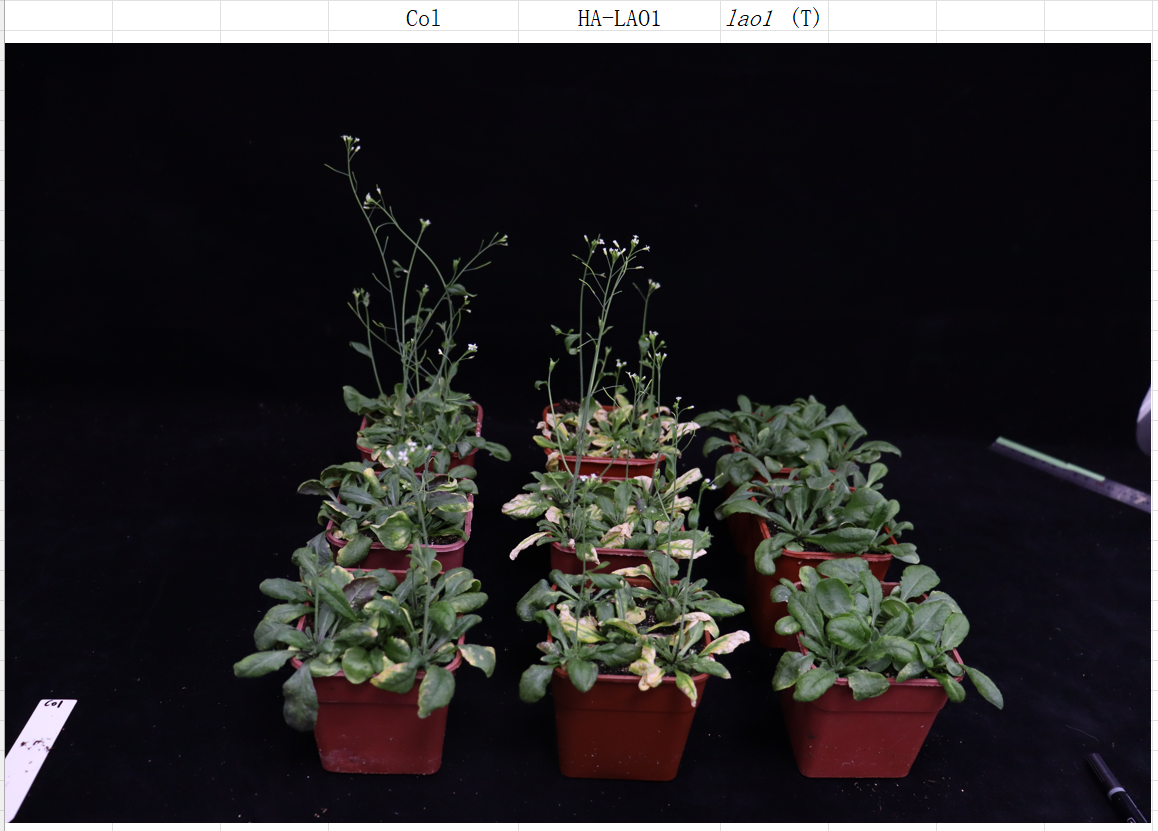

Supplement: Supplementary file 4 — Source data Fig. 1 [file 44319_2025_491_MOESM4_ESM.zip › Figure 1/Figure 1C/Figure 1C.png]

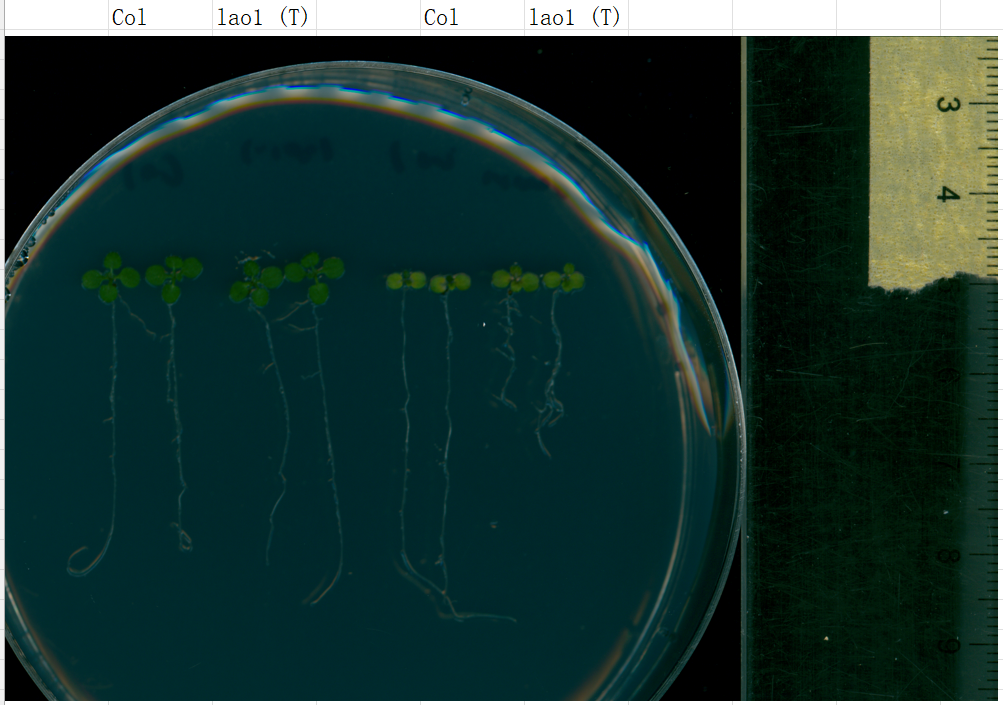

Supplement: Supplementary file 4 — Source data Fig. 1 [file 44319_2025_491_MOESM4_ESM.zip › Figure 1/Figure 1D/Figure 1D.png]

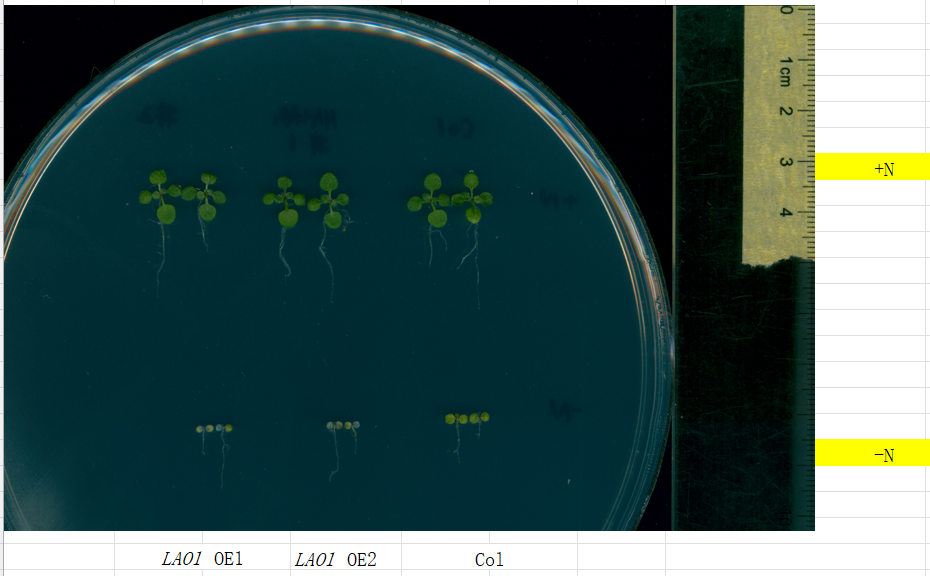

Supplement: Supplementary file 4 — Source data Fig. 1 [file 44319_2025_491_MOESM4_ESM.zip › Figure 1/Figure 1F/Figure 1F.png]

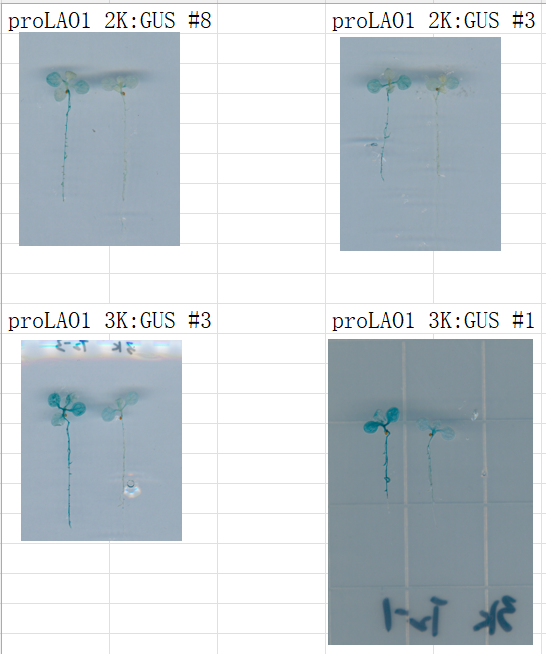

Supplement: Supplementary file 5 — Source data Fig. 2 [file 44319_2025_491_MOESM5_ESM.zip › Figure 2/Figure 2B/Figure 2B.png]

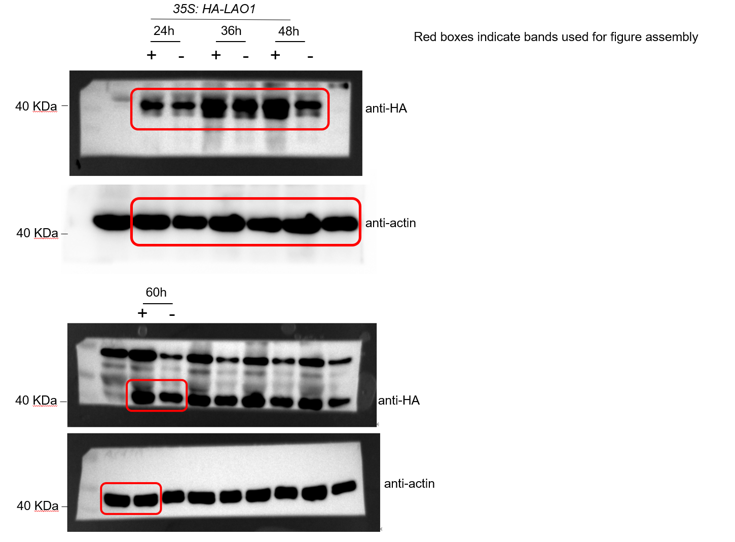

Supplement: Supplementary file 5 — Source data Fig. 2 [file 44319_2025_491_MOESM5_ESM.zip › Figure 2/Figure 2C/图片1.png]

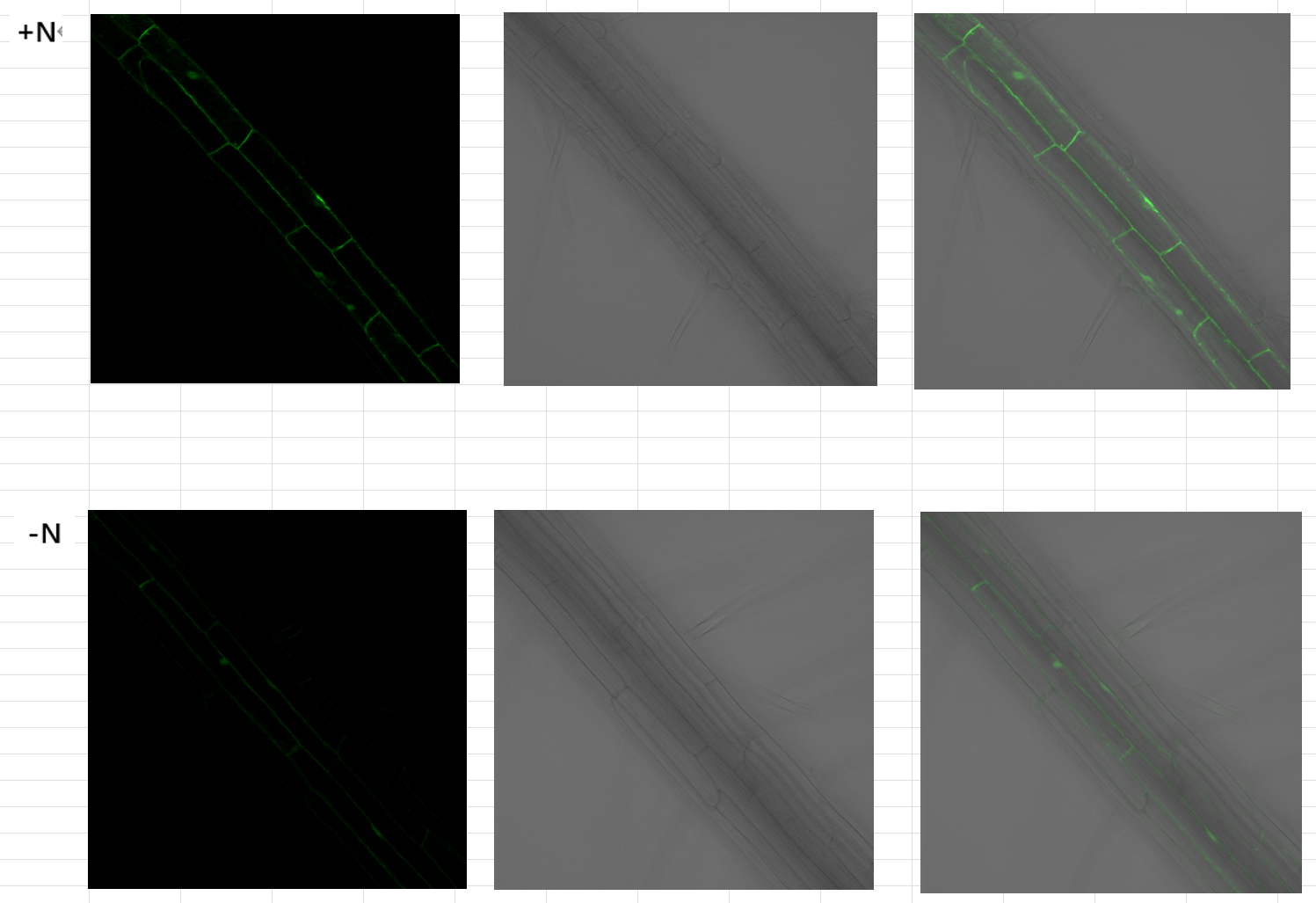

Supplement: Supplementary file 5 — Source data Fig. 2 [file 44319_2025_491_MOESM5_ESM.zip › Figure 2/Figure 2F/Figure 2F.png]

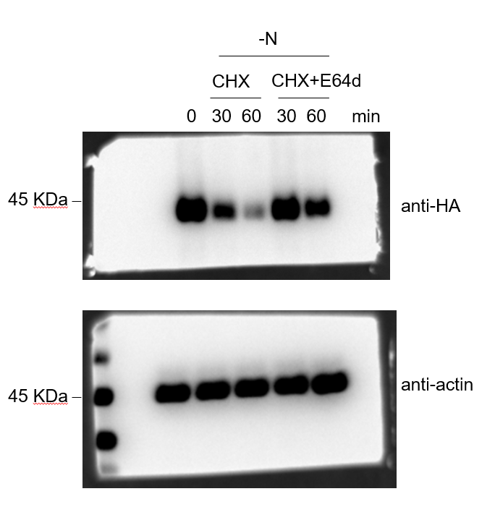

Supplement: Supplementary file 6 — Source data Fig. 3 [file 44319_2025_491_MOESM6_ESM.zip › Figure 3/3A/Figure 3A.png]

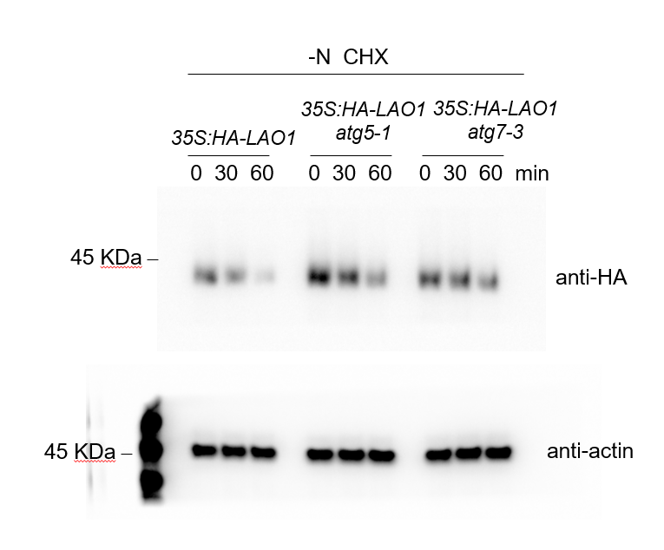

Supplement: Supplementary file 6 — Source data Fig. 3 [file 44319_2025_491_MOESM6_ESM.zip › Figure 3/3B/Figure 3B.png]

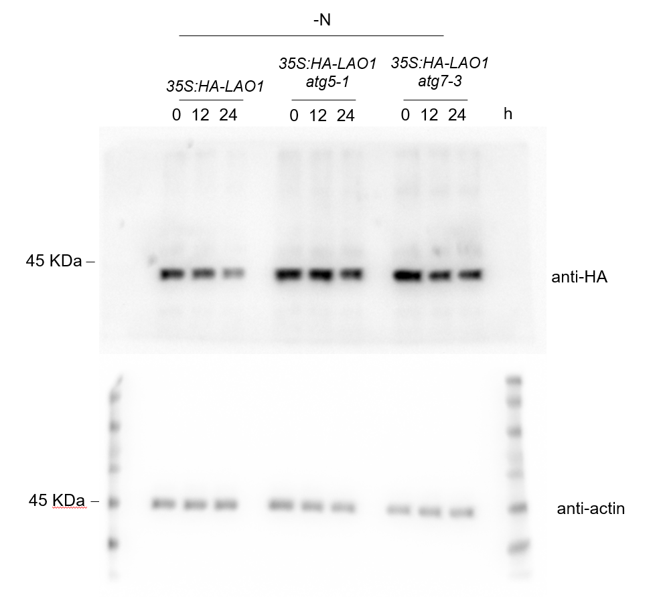

Supplement: Supplementary file 6 — Source data Fig. 3 [file 44319_2025_491_MOESM6_ESM.zip › Figure 3/3C/Figure 3C.png]

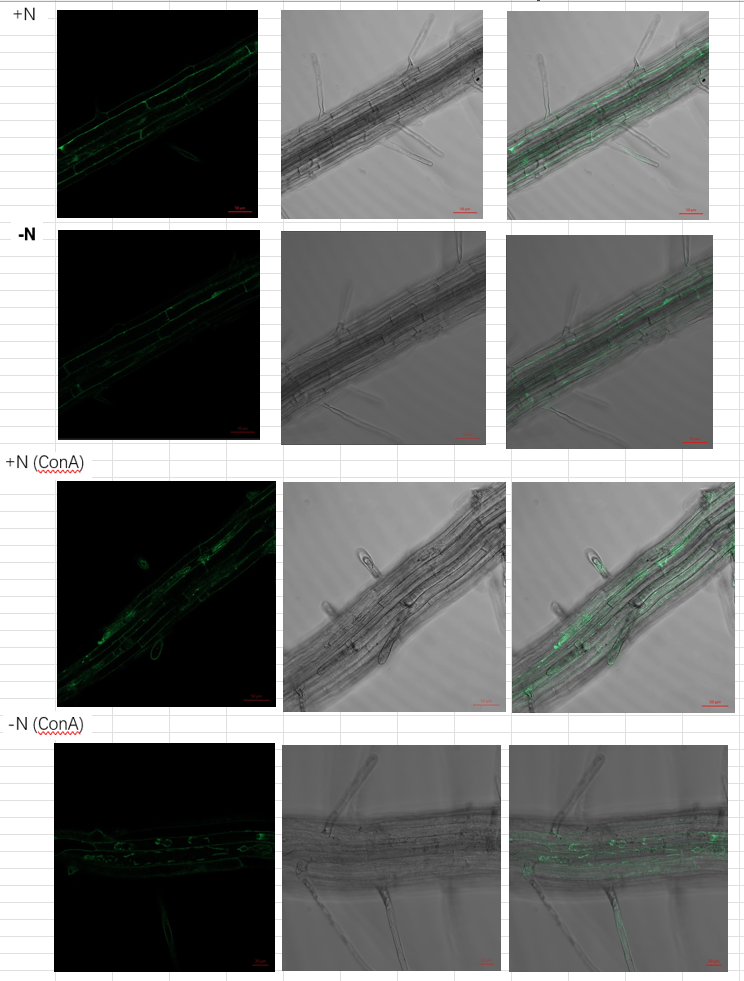

Supplement: Supplementary file 6 — Source data Fig. 3 [file 44319_2025_491_MOESM6_ESM.zip › Figure 3/3E/Figure 3E.png]

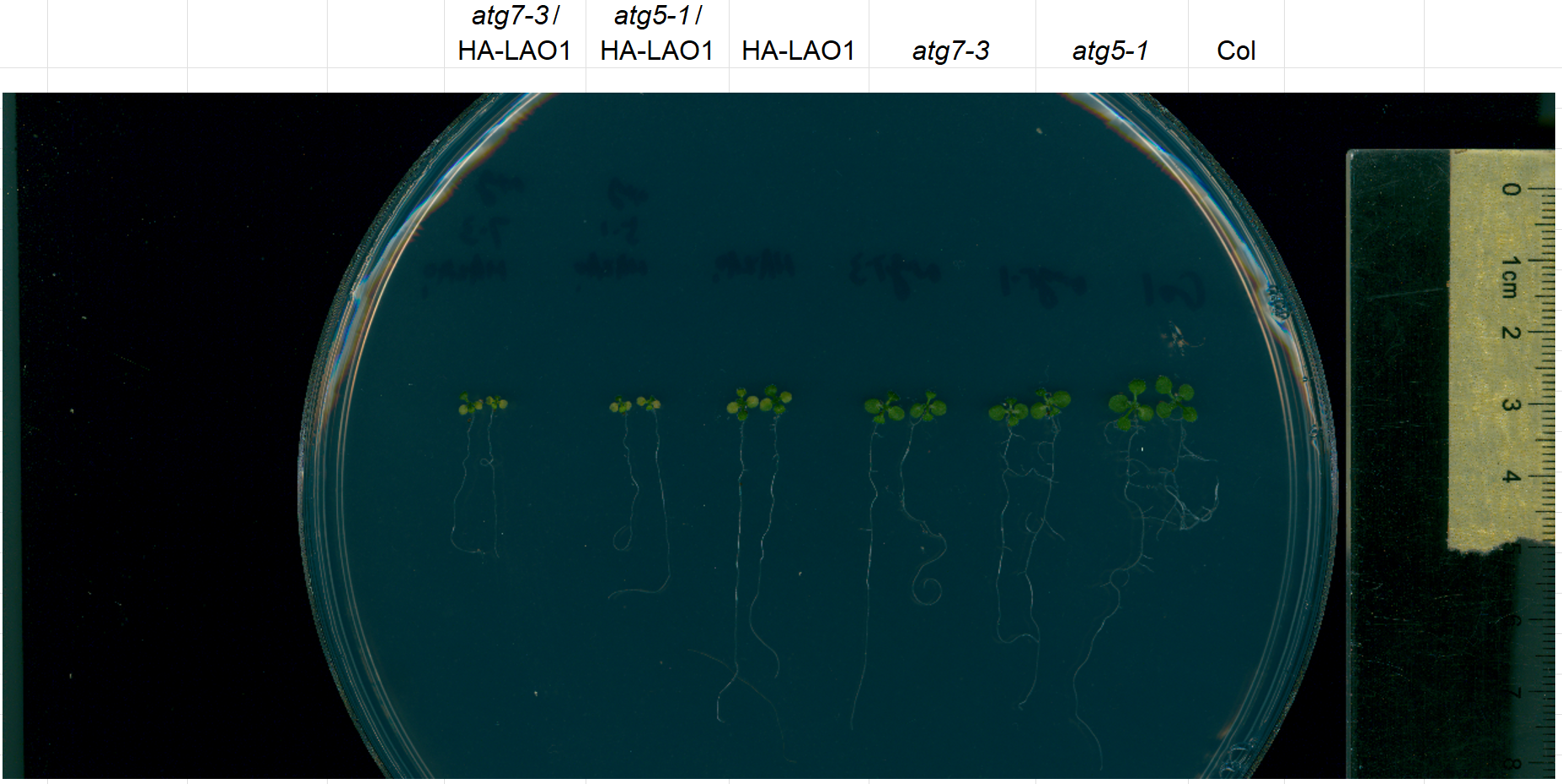

Supplement: Supplementary file 6 — Source data Fig. 3 [file 44319_2025_491_MOESM6_ESM.zip › Figure 3/3F/Figure 3F.png]

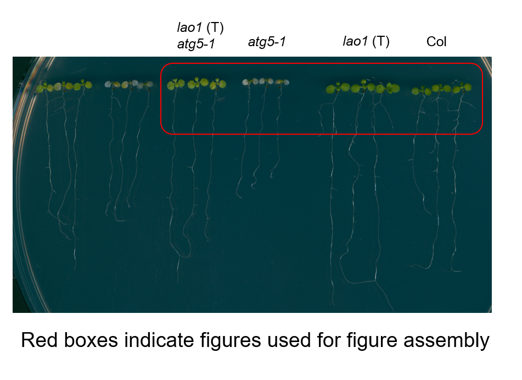

Supplement: Supplementary file 6 — Source data Fig. 3 [file 44319_2025_491_MOESM6_ESM.zip › Figure 3/3G/Figure 3G.png]

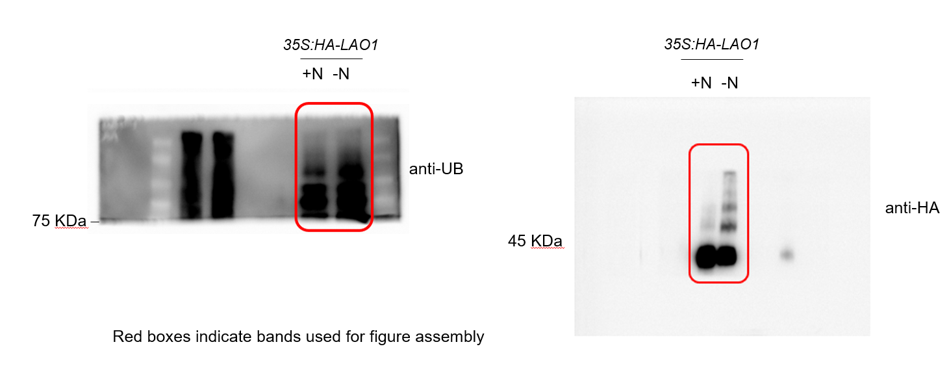

Supplement: Supplementary file 7 — Source data Fig. 4 [file 44319_2025_491_MOESM7_ESM.zip › Figure 4/4A/Figure 4A.png]

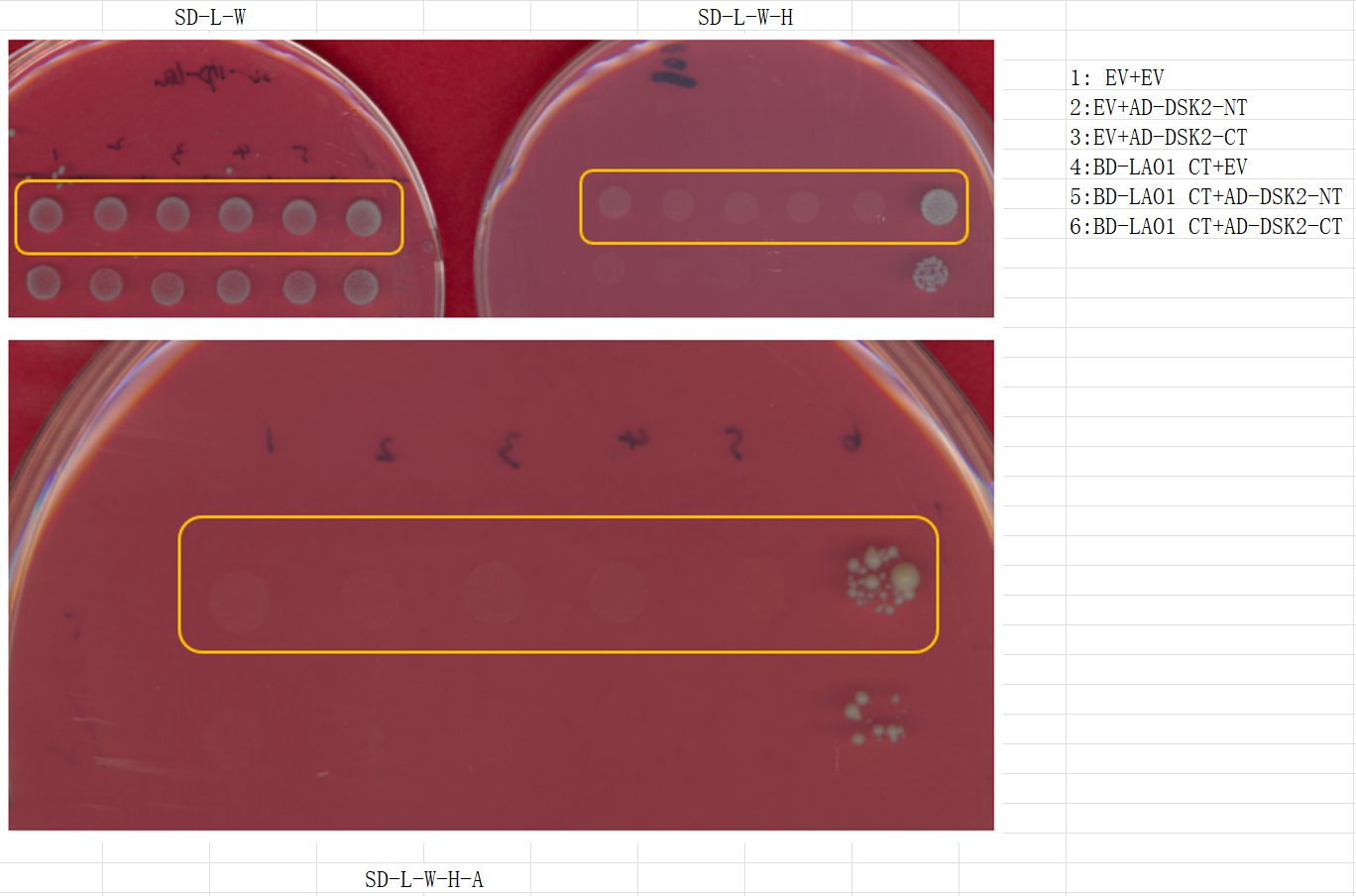

Supplement: Supplementary file 7 — Source data Fig. 4 [file 44319_2025_491_MOESM7_ESM.zip › Figure 4/4B/Figure 4B.png]

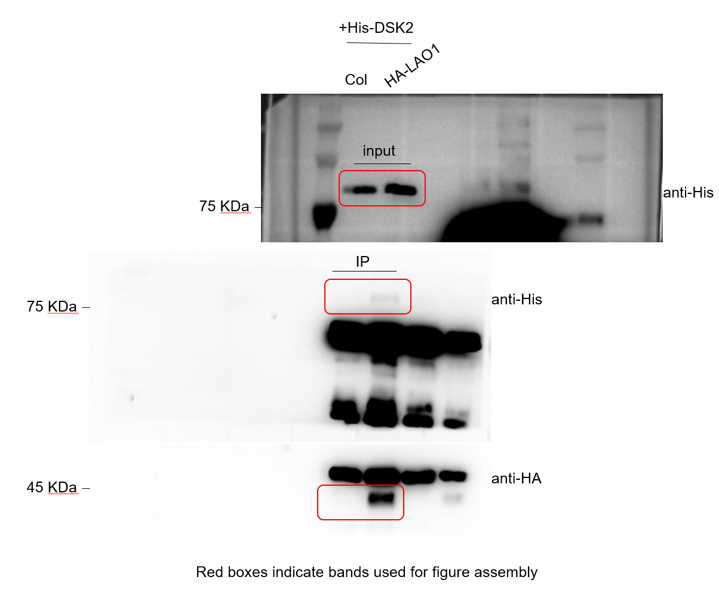

Supplement: Supplementary file 7 — Source data Fig. 4 [file 44319_2025_491_MOESM7_ESM.zip › Figure 4/4C/Figure 4C.png]

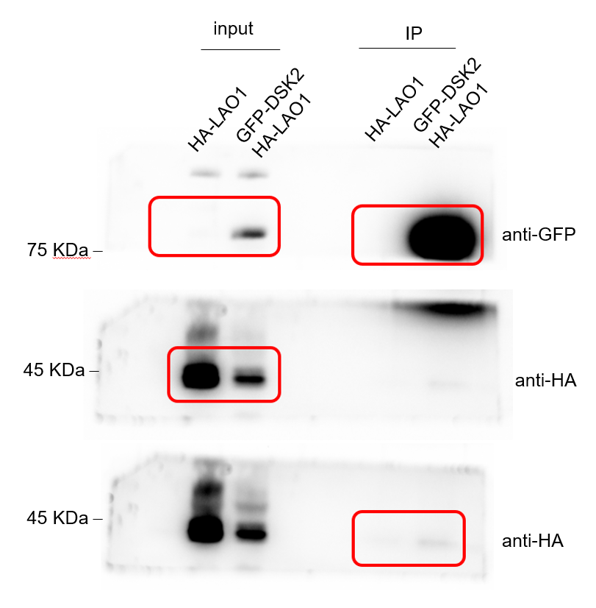

Supplement: Supplementary file 7 — Source data Fig. 4 [file 44319_2025_491_MOESM7_ESM.zip › Figure 4/4D/Figure 4D.png]

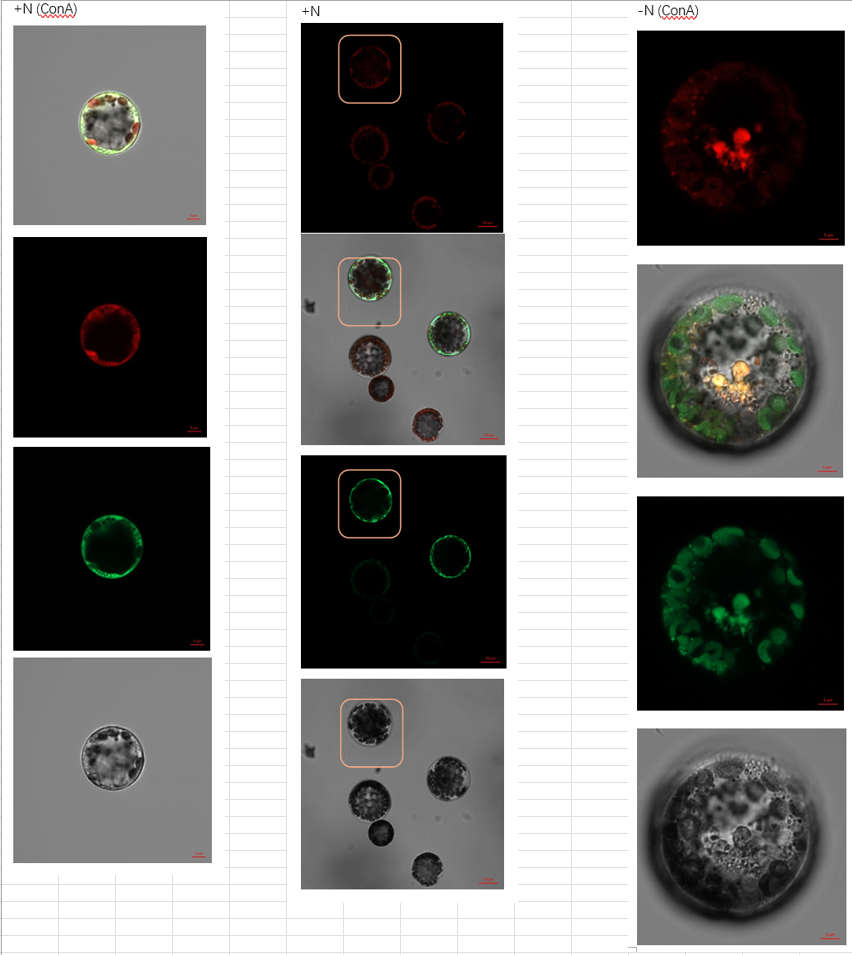

Supplement: Supplementary file 7 — Source data Fig. 4 [file 44319_2025_491_MOESM7_ESM.zip › Figure 4/4E/Figure 4E.png]

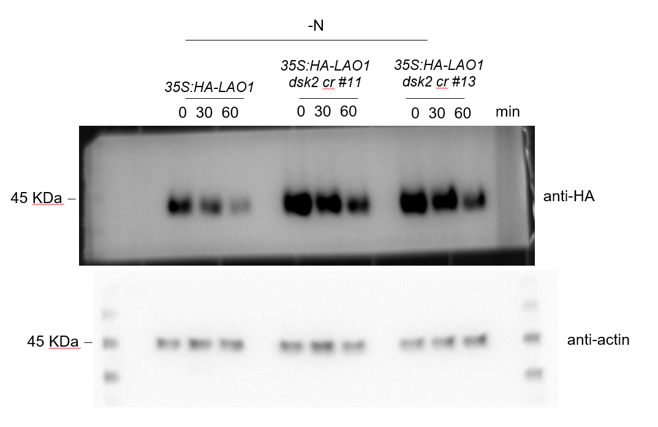

Supplement: Supplementary file 7 — Source data Fig. 4 [file 44319_2025_491_MOESM7_ESM.zip › Figure 4/4F/Figure 4F.png]

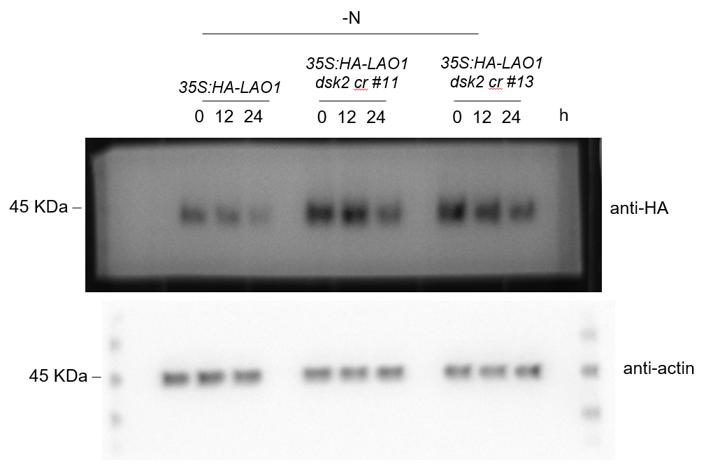

Supplement: Supplementary file 7 — Source data Fig. 4 [file 44319_2025_491_MOESM7_ESM.zip › Figure 4/4G/Figure 4G.png]

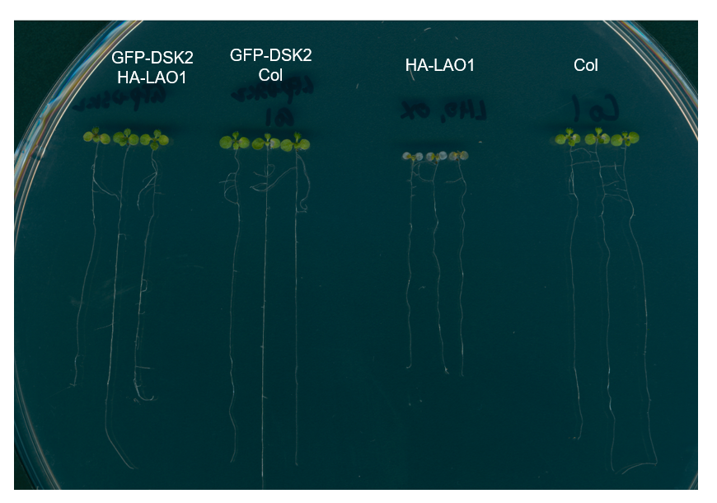

Supplement: Supplementary file 8 — Source data Fig. 5 [file 44319_2025_491_MOESM8_ESM.zip › Figure 5/5A/Figure 5A.png]

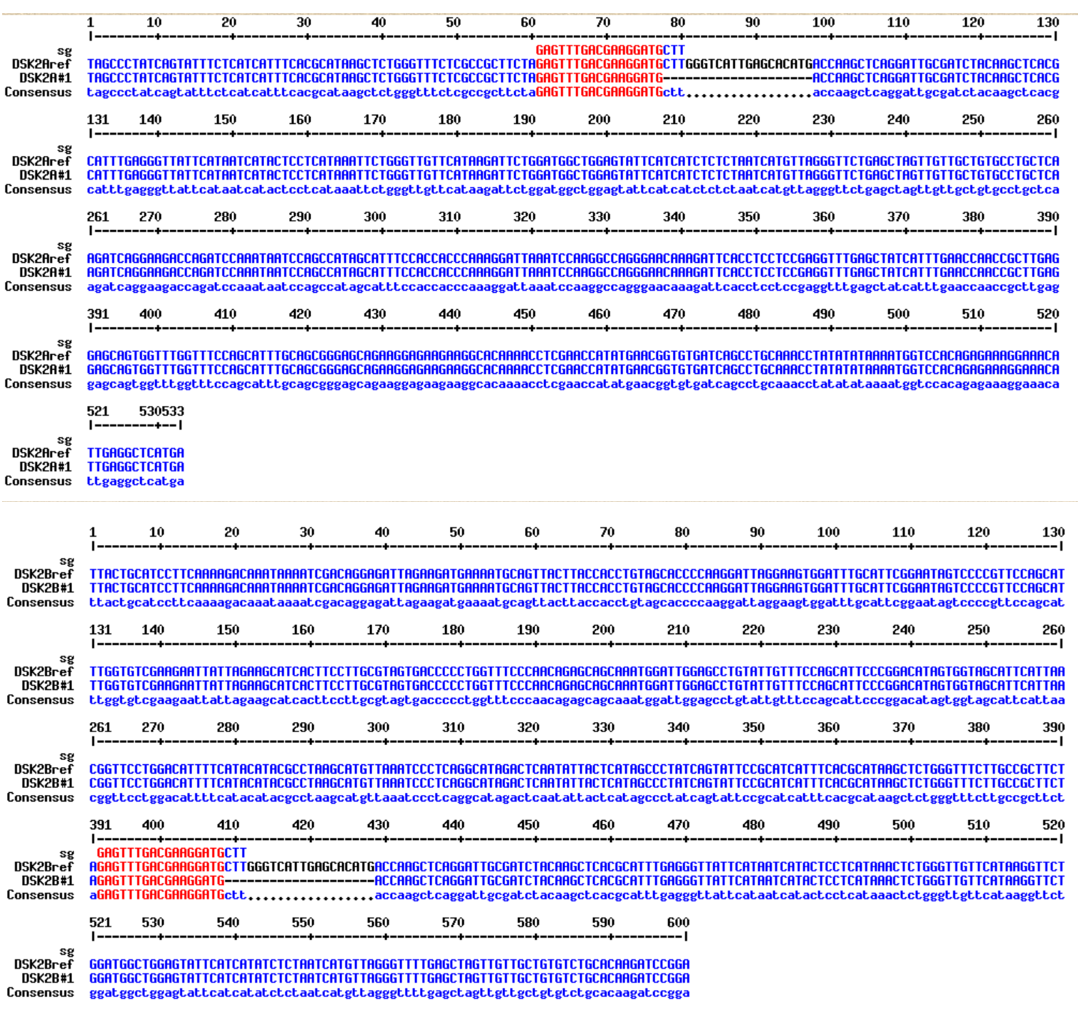

Supplement: Supplementary file 8 — Source data Fig. 5 [file 44319_2025_491_MOESM8_ESM.zip › Figure 5/5C/Figure 5C#1.png]

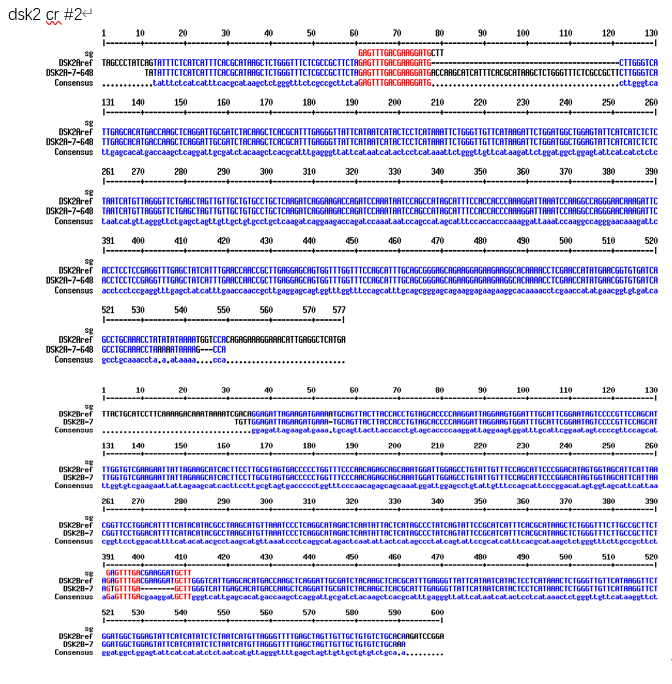

Supplement: Supplementary file 8 — Source data Fig. 5 [file 44319_2025_491_MOESM8_ESM.zip › Figure 5/5C/Figure 5C#2.png]

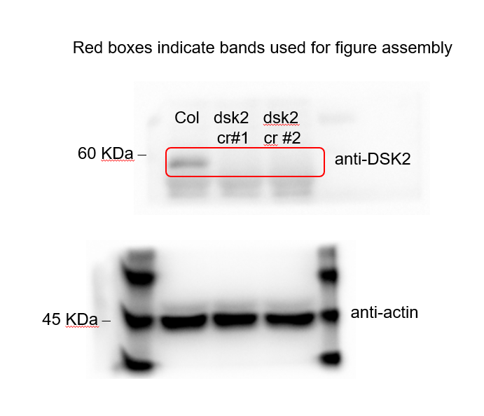

Supplement: Supplementary file 8 — Source data Fig. 5 [file 44319_2025_491_MOESM8_ESM.zip › Figure 5/5D/Figure 5D.png]

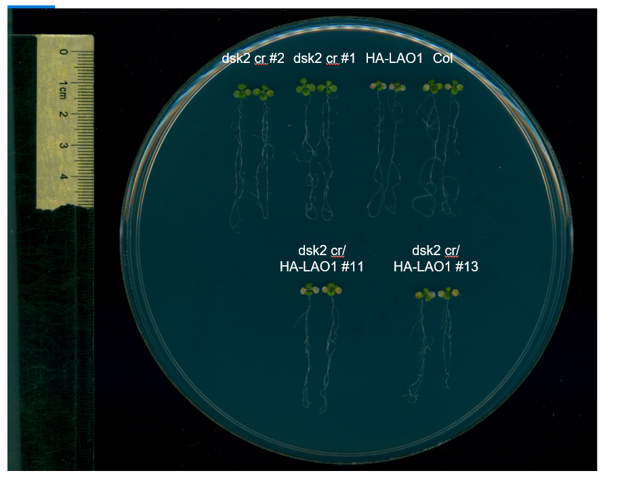

Supplement: Supplementary file 8 — Source data Fig. 5 [file 44319_2025_491_MOESM8_ESM.zip › Figure 5/5E/Figure 5E.png]

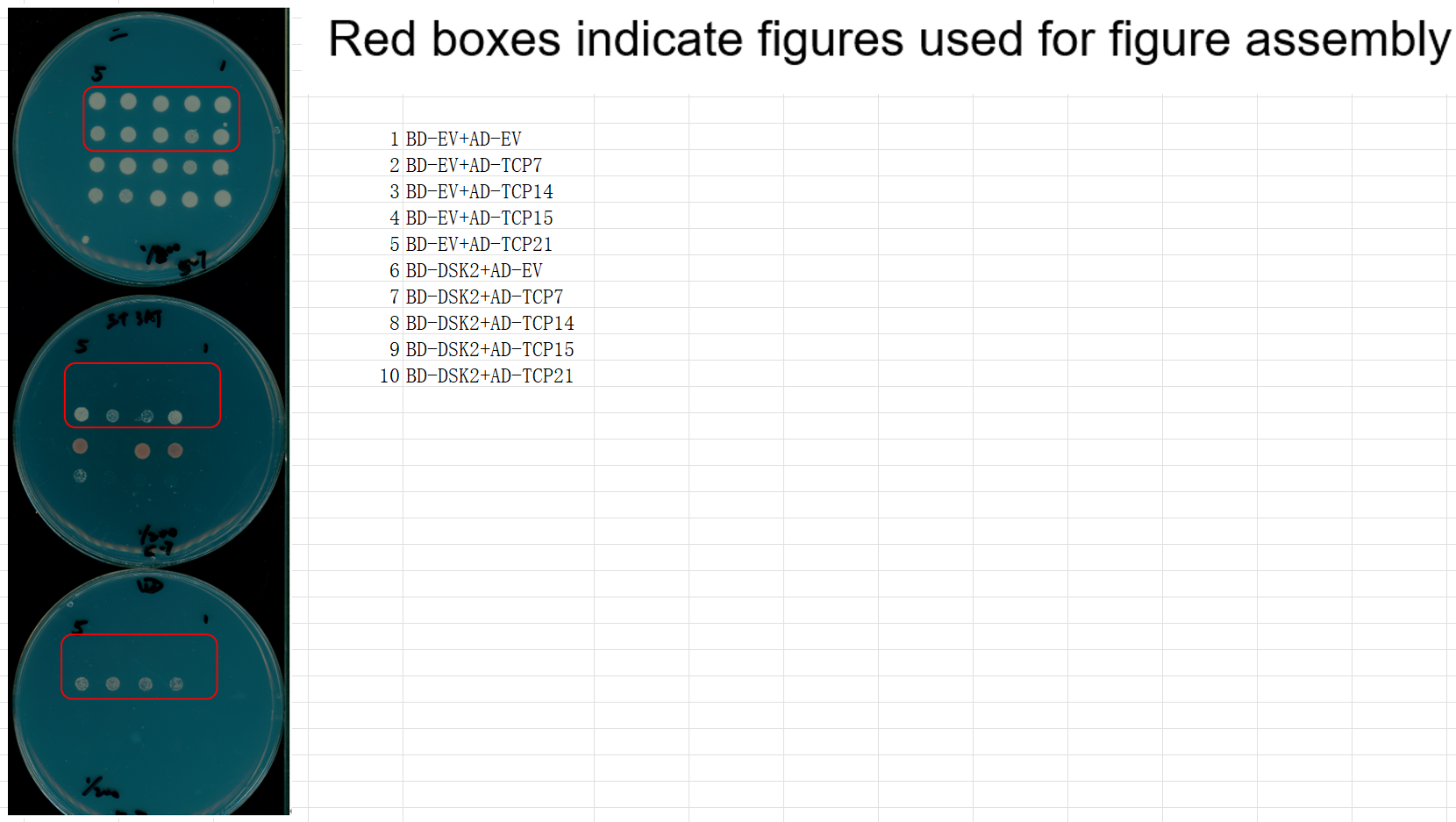

Supplement: Supplementary file 9 — Source data Fig. 6 [file 44319_2025_491_MOESM9_ESM.zip › Figure 6/6A/Figure 6A.png]

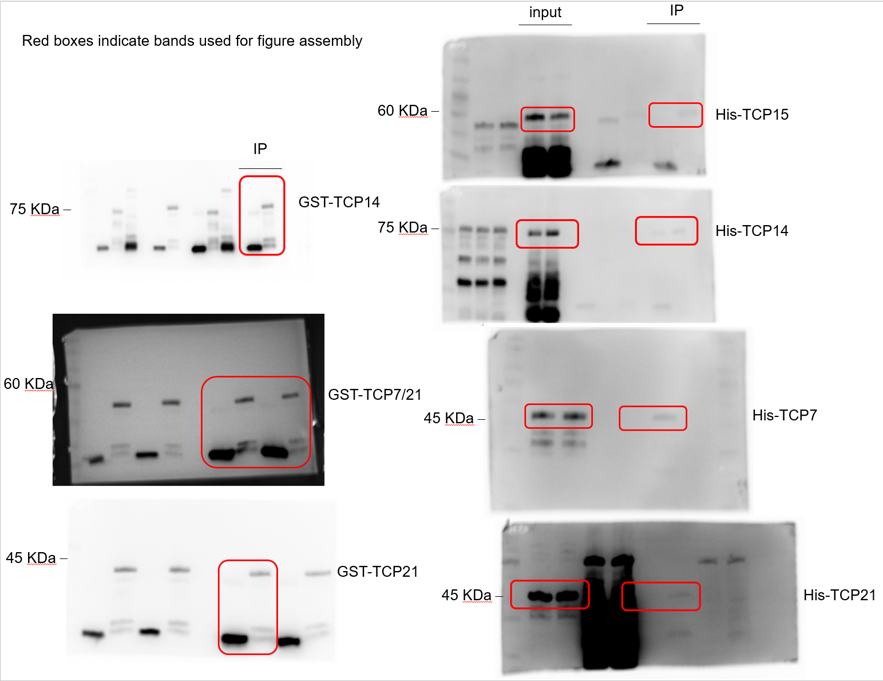

Supplement: Supplementary file 9 — Source data Fig. 6 [file 44319_2025_491_MOESM9_ESM.zip › Figure 6/6B/Figure 6B.png]

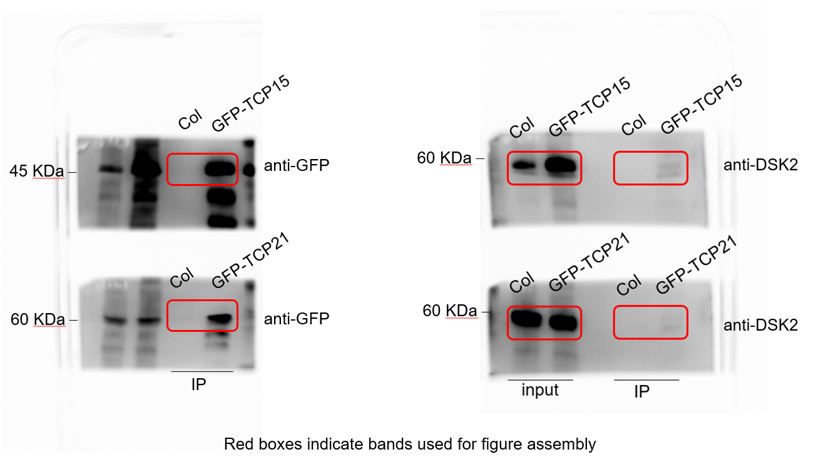

Supplement: Supplementary file 9 — Source data Fig. 6 [file 44319_2025_491_MOESM9_ESM.zip › Figure 6/6C/Figure 6C.png]

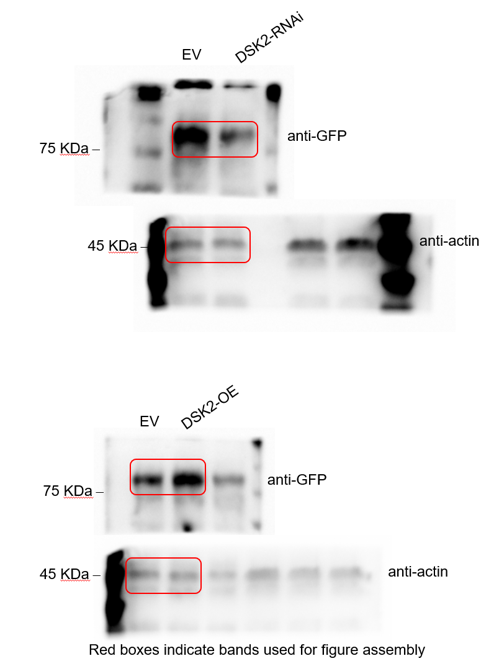

Supplement: Supplementary file 9 — Source data Fig. 6 [file 44319_2025_491_MOESM9_ESM.zip › Figure 6/6D/Figure 6D.png]

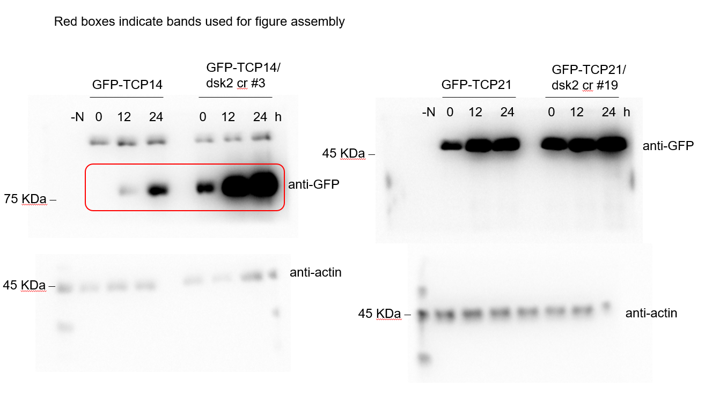

Supplement: Supplementary file 9 — Source data Fig. 6 [file 44319_2025_491_MOESM9_ESM.zip › Figure 6/6G/Figure 6G.png]

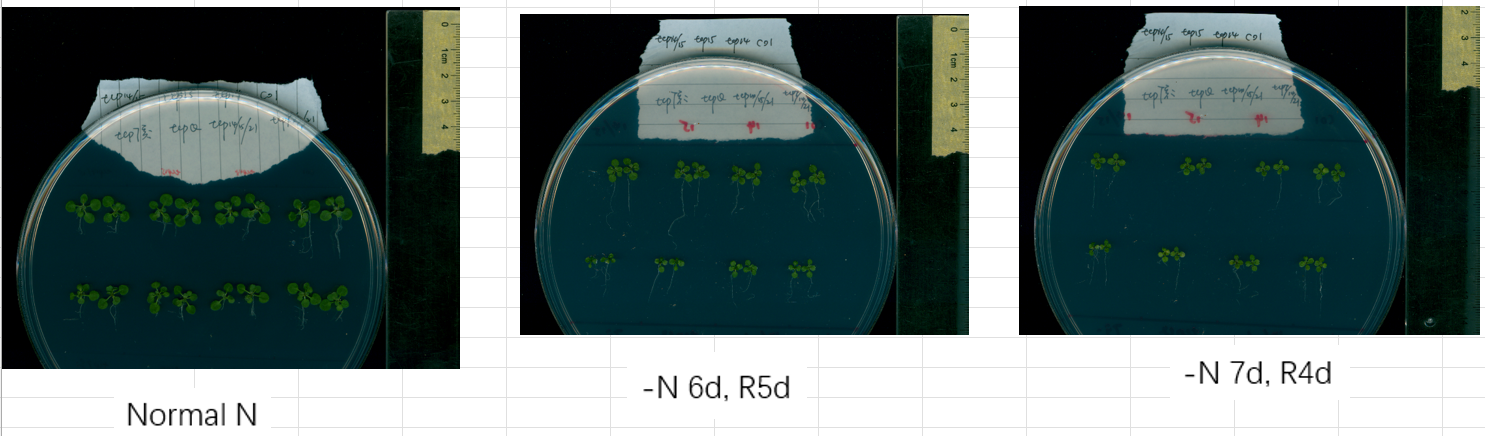

Supplement: Supplementary file 10 — Source data Fig. 7 [file 44319_2025_491_MOESM10_ESM.zip › Figure 7/Figure 7A/Figure 7A.png]

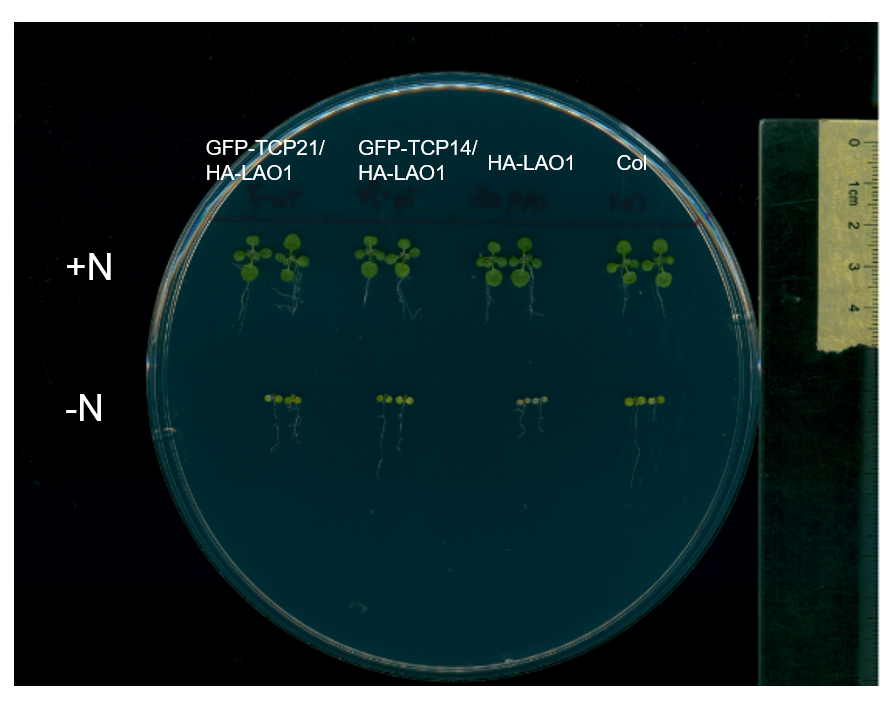

Supplement: Supplementary file 10 — Source data Fig. 7 [file 44319_2025_491_MOESM10_ESM.zip › Figure 7/Figure 7C/Figure 7C.png]

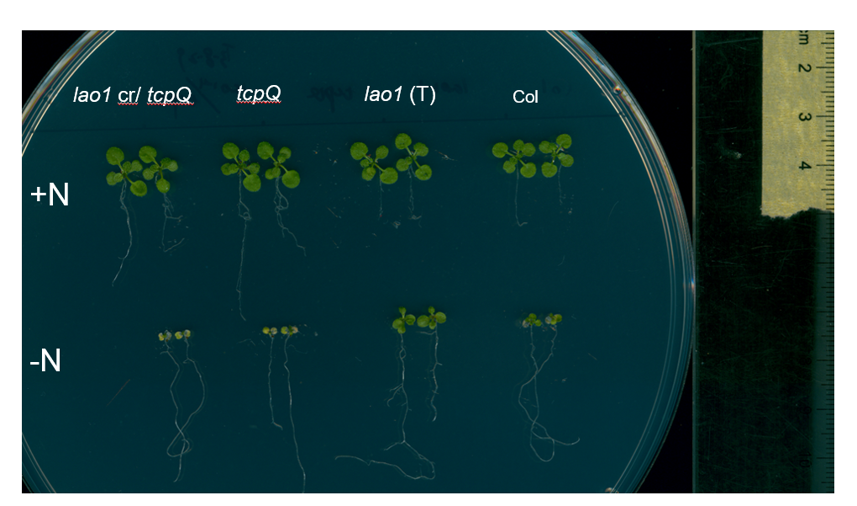

Supplement: Supplementary file 10 — Source data Fig. 7 [file 44319_2025_491_MOESM10_ESM.zip › Figure 7/Figure 7E/Figure 7E.png]

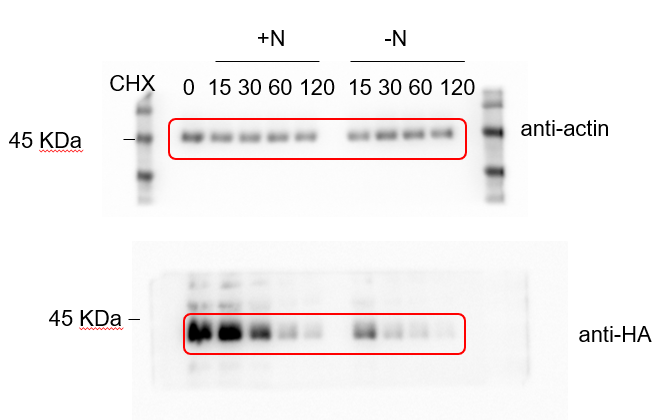

Supplement: Supplementary file 11 — EV+Appendix Fig Source Data [file 44319_2025_491_MOESM11_ESM.zip › Appendix/Appendix Figure S2/Appendix Figure S2A.png]

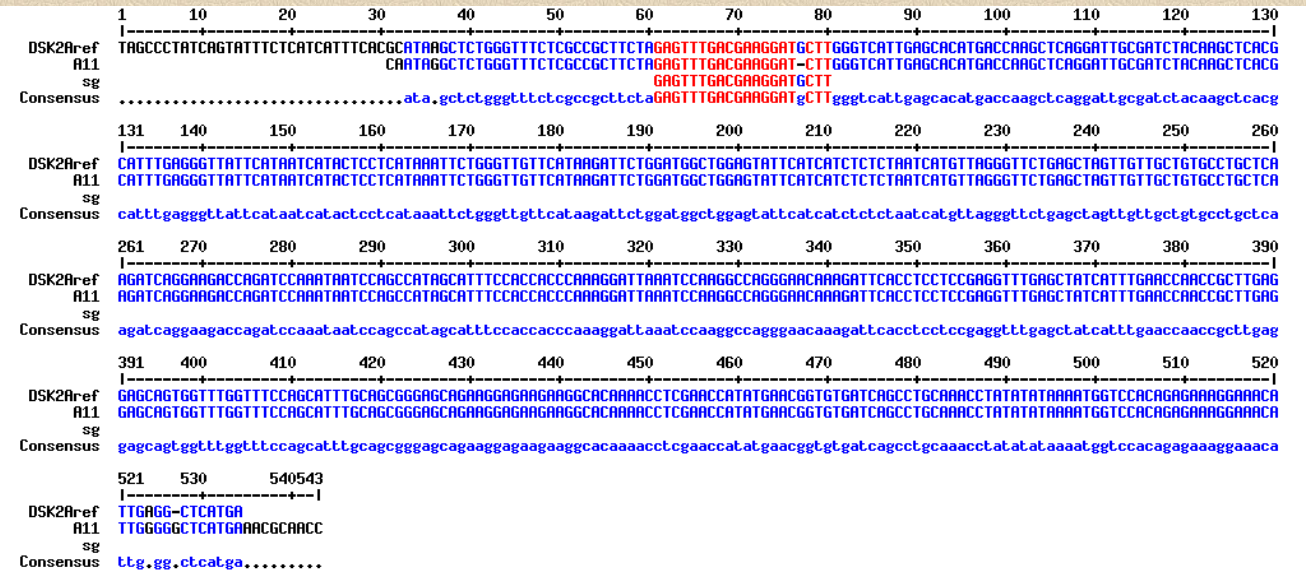

Supplement: Supplementary file 11 — EV+Appendix Fig Source Data [file 44319_2025_491_MOESM11_ESM.zip › Appendix/Appendix Figure S4/Appendix Figure S3#11A.png]

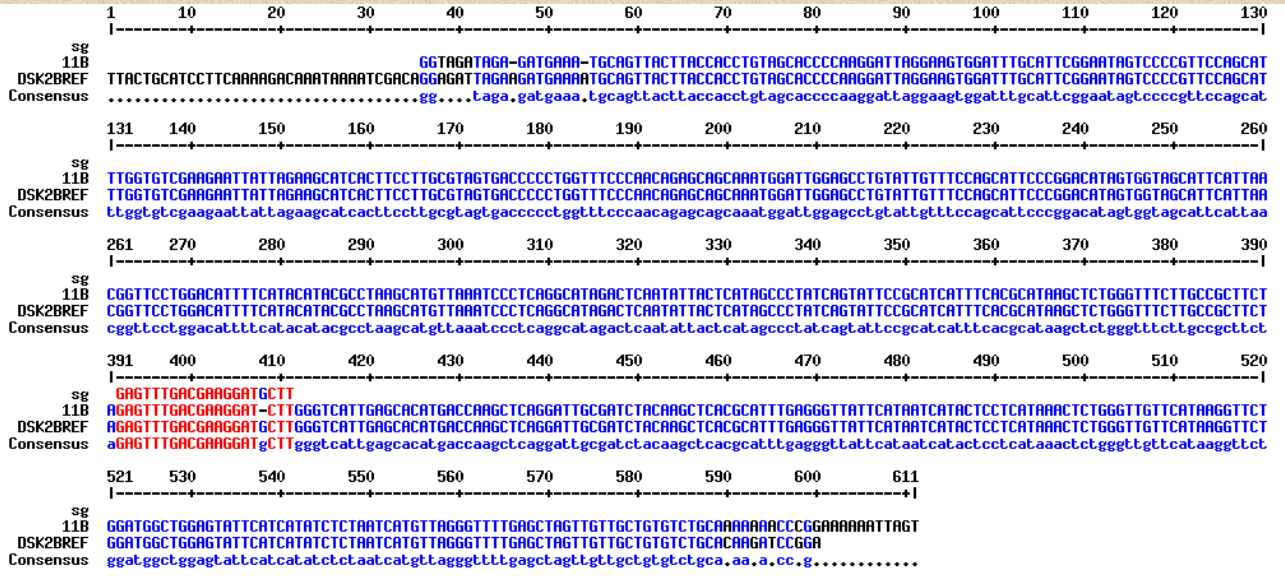

Supplement: Supplementary file 11 — EV+Appendix Fig Source Data [file 44319_2025_491_MOESM11_ESM.zip › Appendix/Appendix Figure S4/Appendix Figure S3#11B.png]

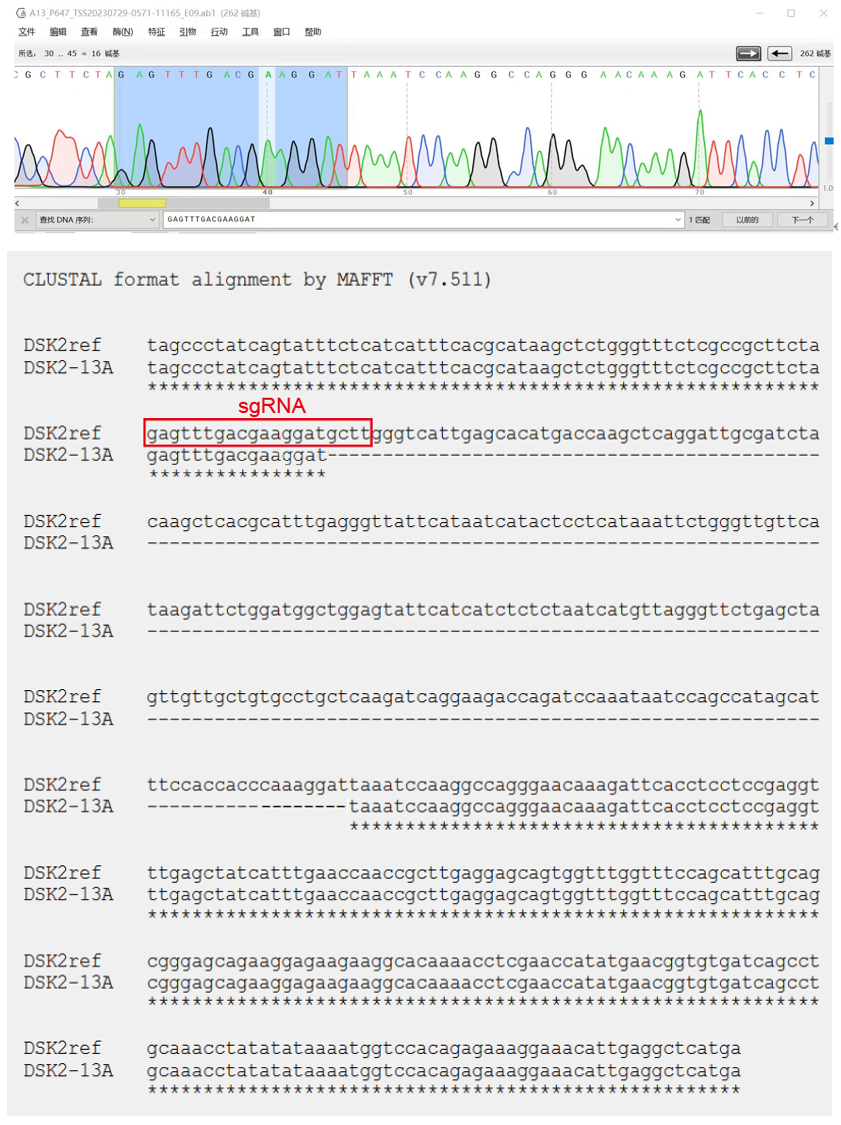

Supplement: Supplementary file 11 — EV+Appendix Fig Source Data [file 44319_2025_491_MOESM11_ESM.zip › Appendix/Appendix Figure S4/Appendix Figure S3#13A.png]

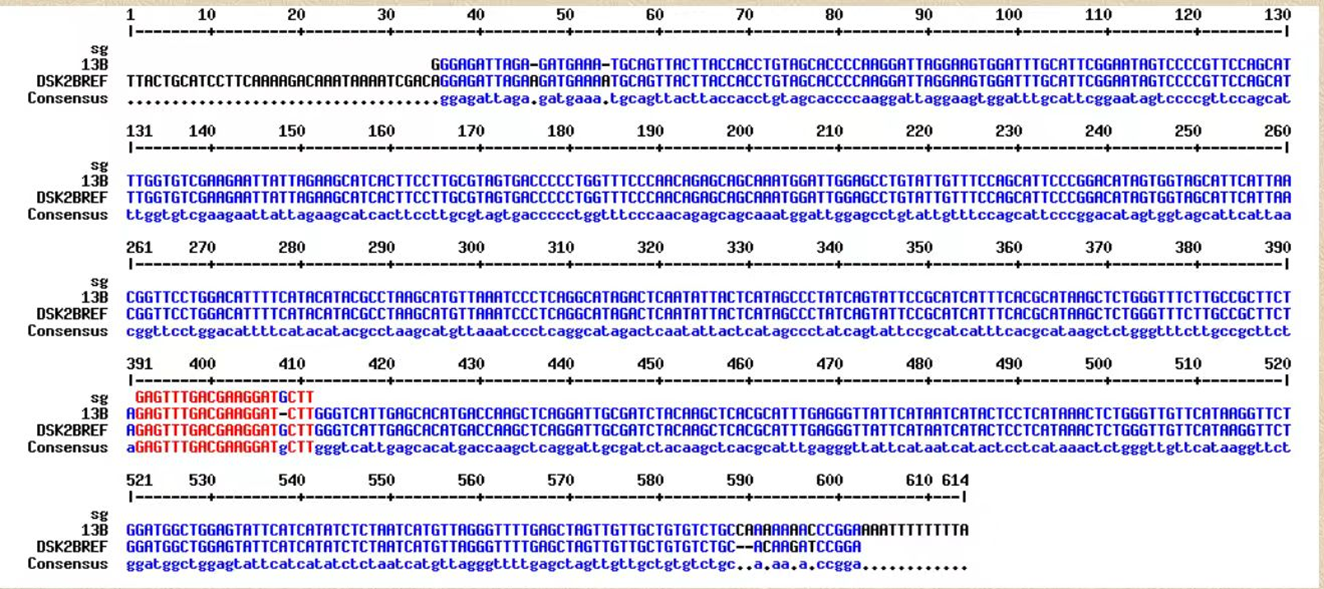

Supplement: Supplementary file 11 — EV+Appendix Fig Source Data [file 44319_2025_491_MOESM11_ESM.zip › Appendix/Appendix Figure S4/Appendix Figure S3#13B.png]

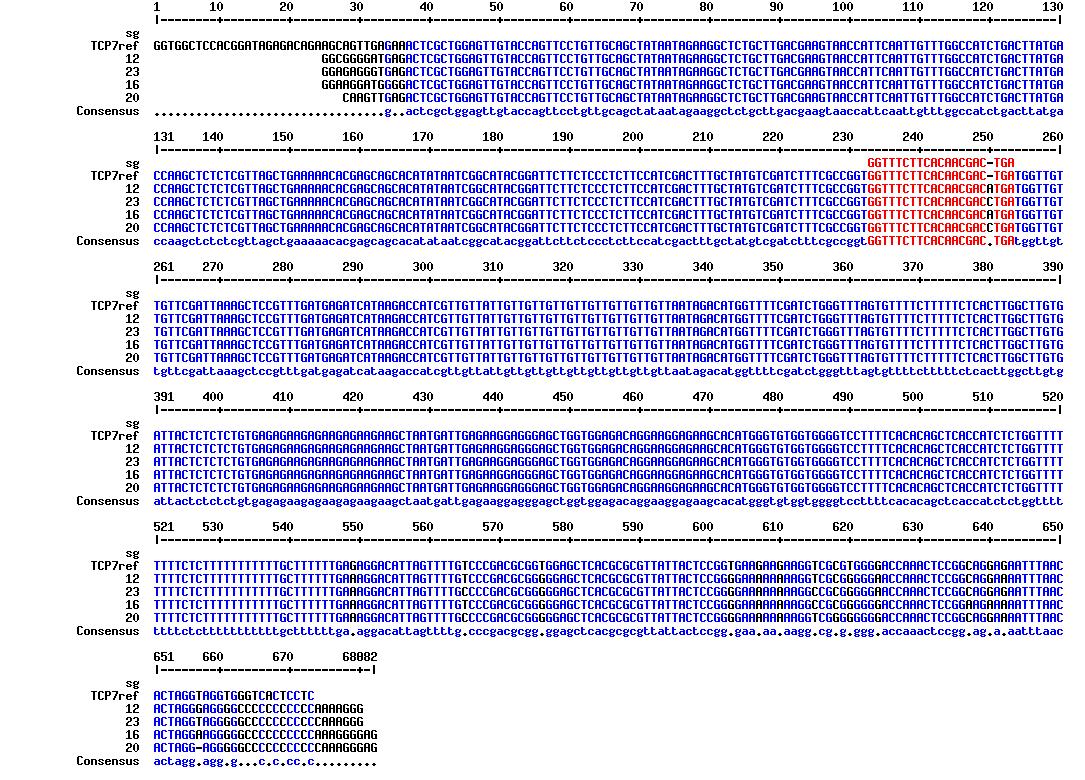

Supplement: Supplementary file 11 — EV+Appendix Fig Source Data [file 44319_2025_491_MOESM11_ESM.zip › Appendix/Appendix Figure S5/Appendix Figure S4-1.png]

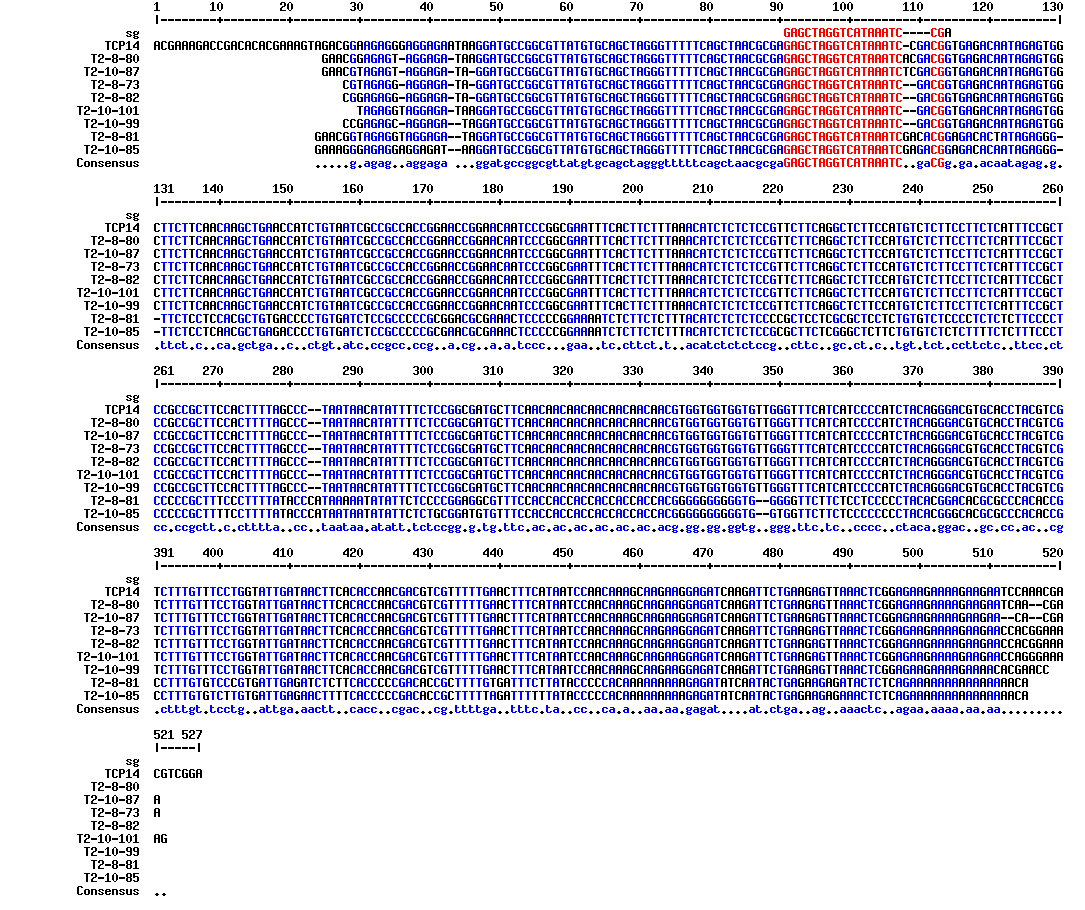

Supplement: Supplementary file 11 — EV+Appendix Fig Source Data [file 44319_2025_491_MOESM11_ESM.zip › Appendix/Appendix Figure S5/Appendix Figure S4-2.png]

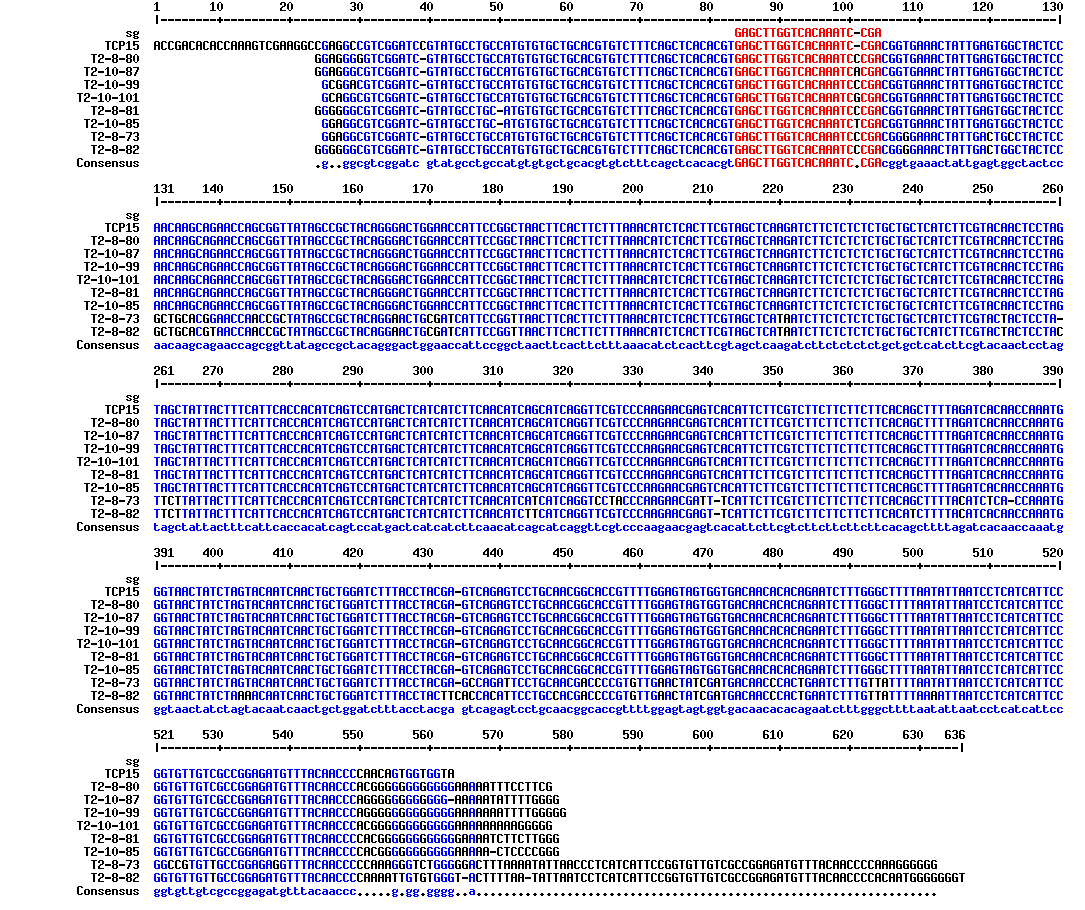

Supplement: Supplementary file 11 — EV+Appendix Fig Source Data [file 44319_2025_491_MOESM11_ESM.zip › Appendix/Appendix Figure S5/Appendix Figure S4-3.png]

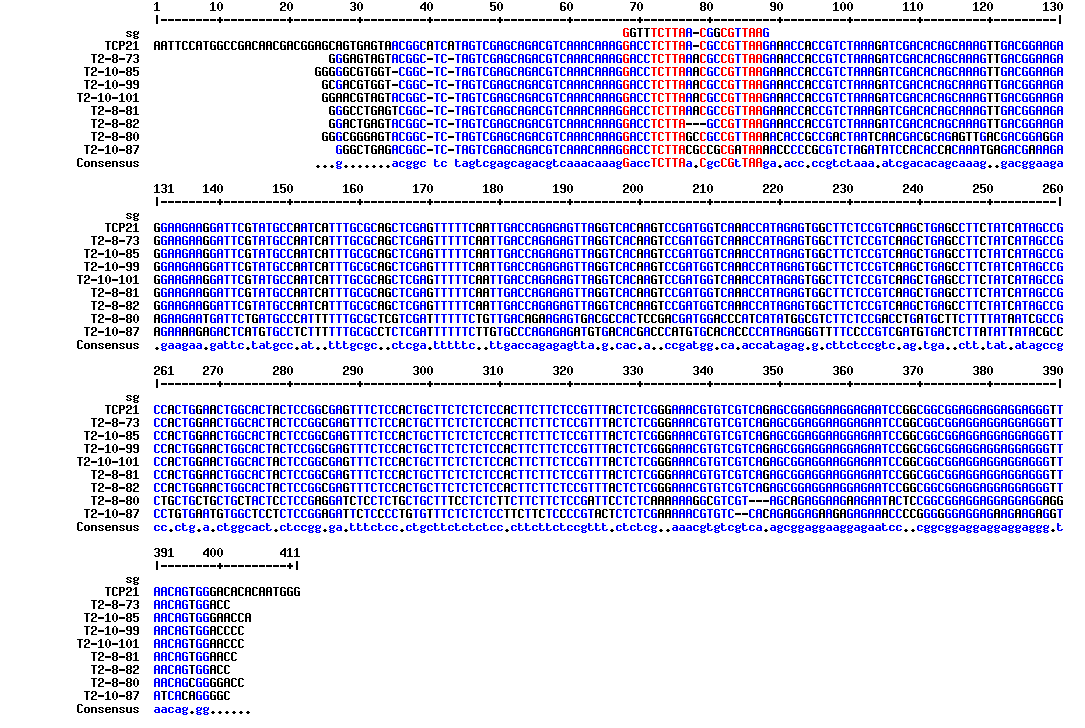

Supplement: Supplementary file 11 — EV+Appendix Fig Source Data [file 44319_2025_491_MOESM11_ESM.zip › Appendix/Appendix Figure S5/Appendix Figure S4-4.png]

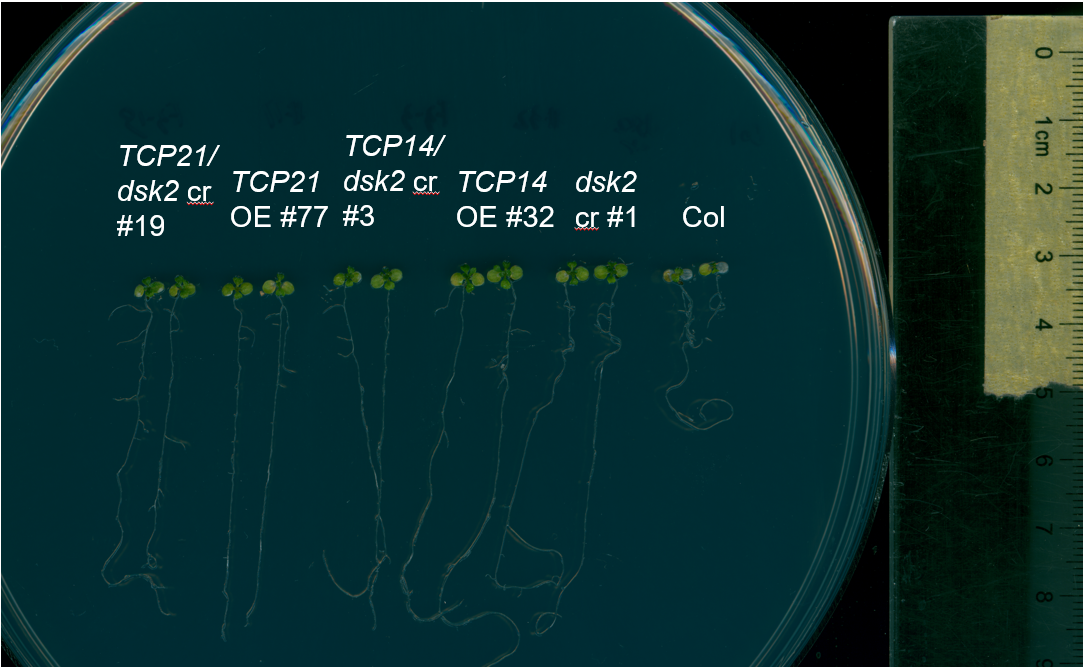

Supplement: Supplementary file 11 — EV+Appendix Fig Source Data [file 44319_2025_491_MOESM11_ESM.zip › Appendix/Appendix Figure S6/Appendix Figure S6B.png]

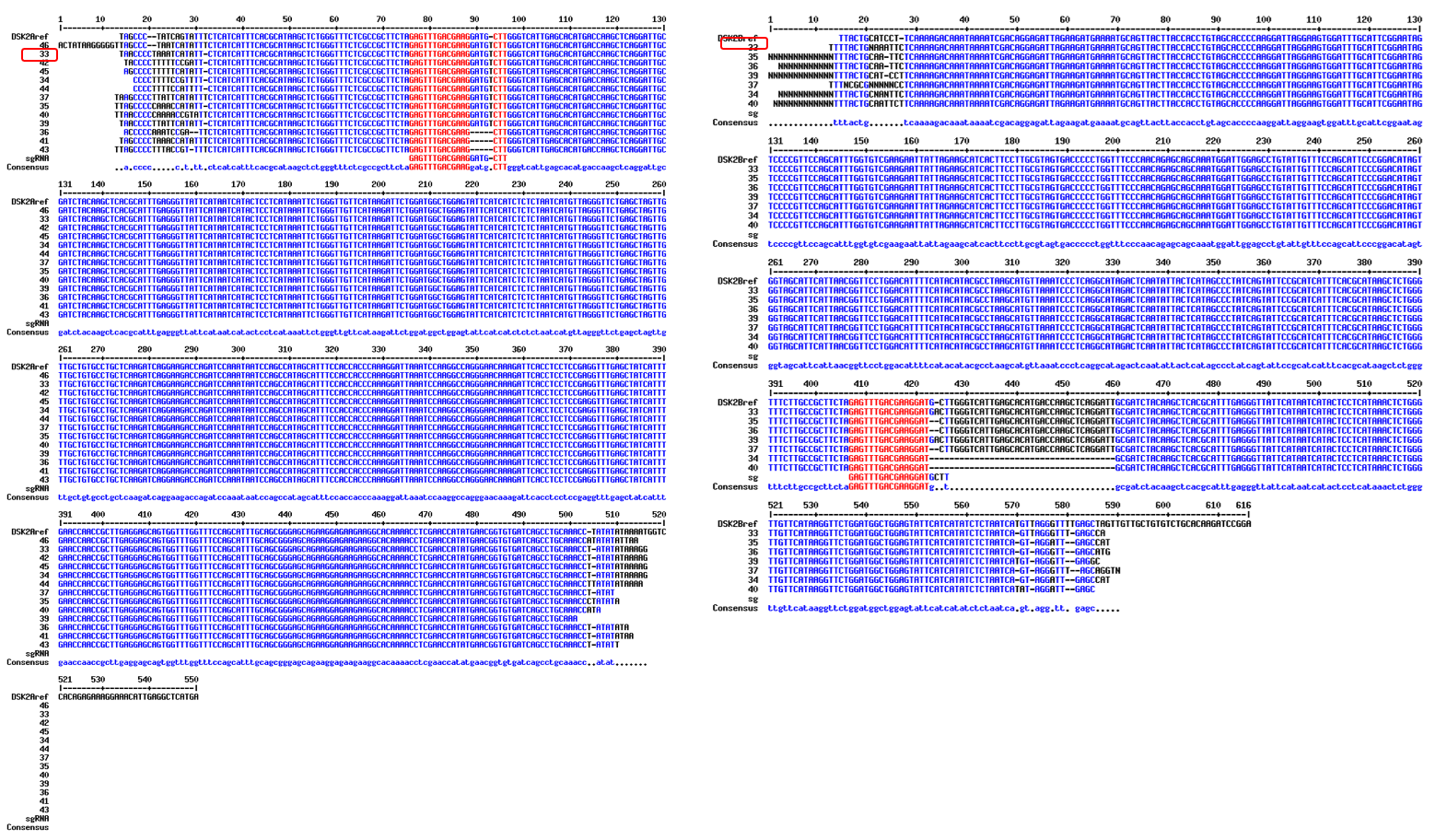

Supplement: Supplementary file 11 — EV+Appendix Fig Source Data [file 44319_2025_491_MOESM11_ESM.zip › Appendix/Appendix Figure S7/Appendix Figure S7A.png]

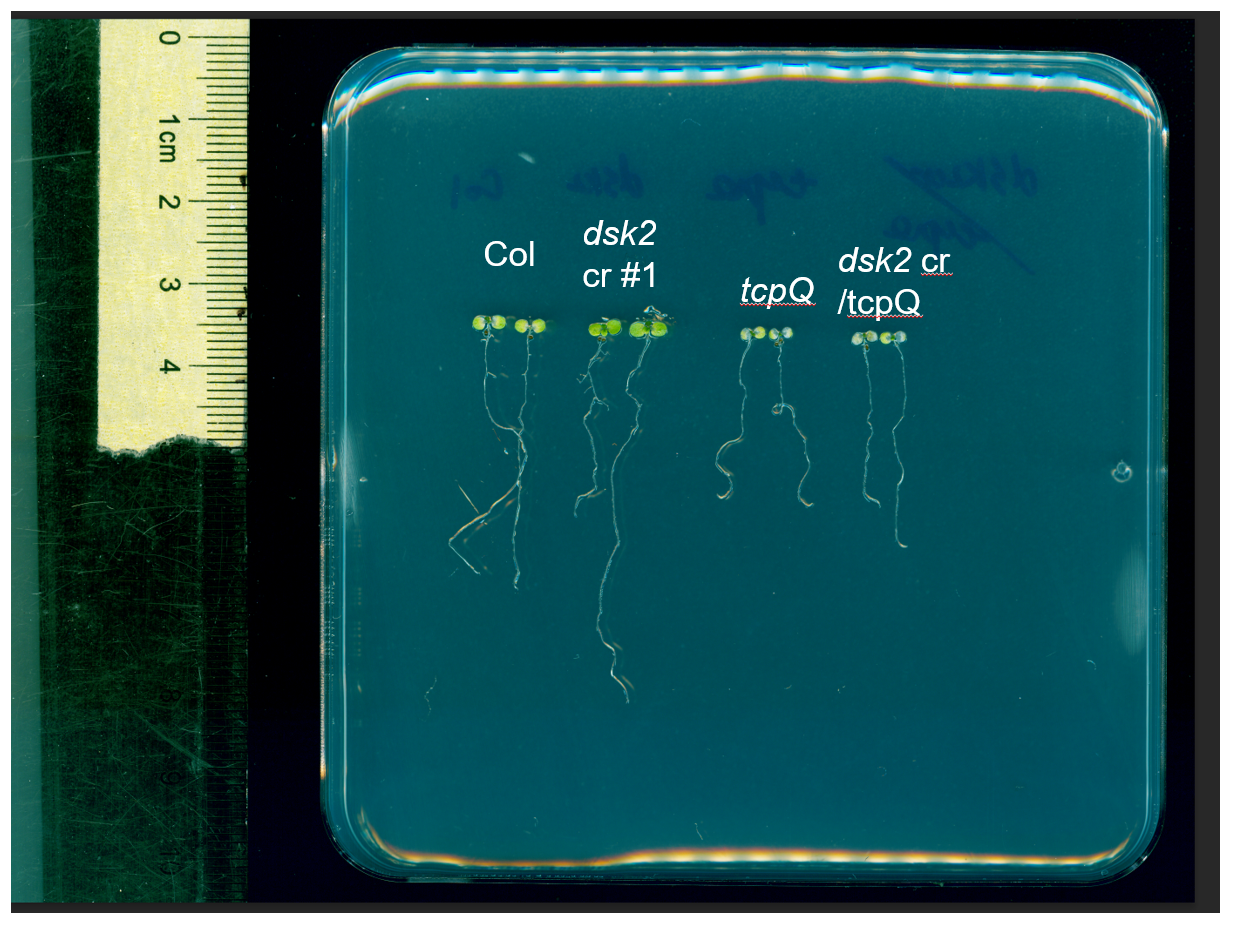

Supplement: Supplementary file 11 — EV+Appendix Fig Source Data [file 44319_2025_491_MOESM11_ESM.zip › Appendix/Appendix Figure S7/Appendix Figure S7B.png]

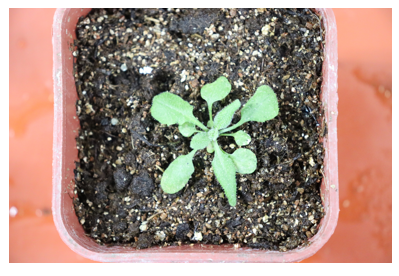

Supplement: Supplementary file 11 — EV+Appendix Fig Source Data [file 44319_2025_491_MOESM11_ESM.zip › EV/EV2/EV2A/EV2A.png]

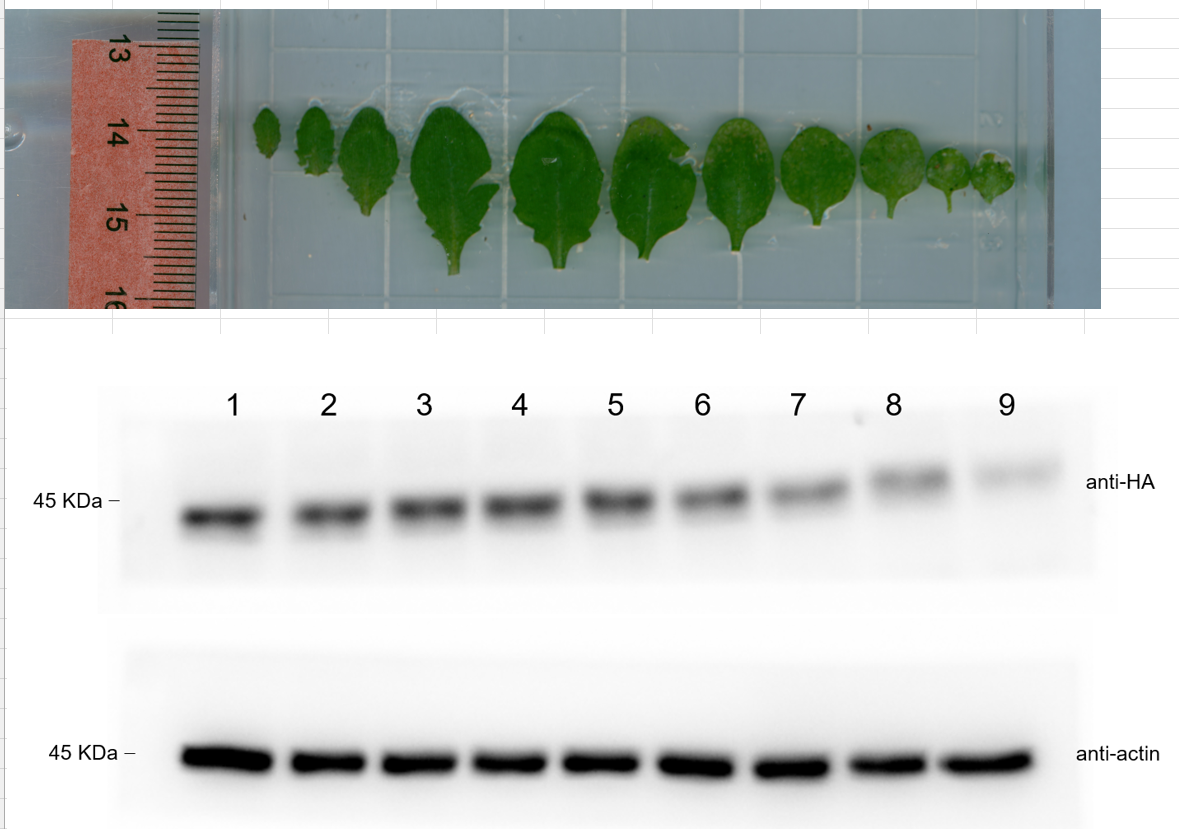

Supplement: Supplementary file 11 — EV+Appendix Fig Source Data [file 44319_2025_491_MOESM11_ESM.zip › EV/EV2/EV2C/EV2C.png]

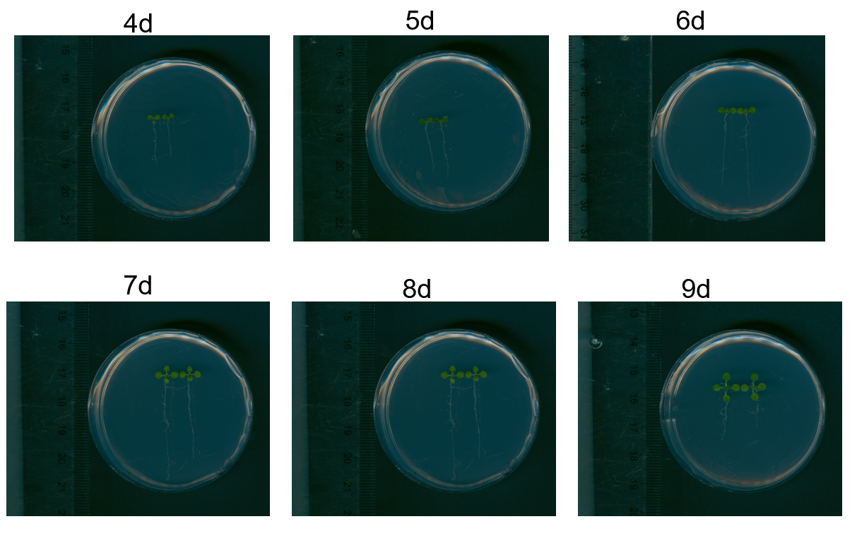

Supplement: Supplementary file 11 — EV+Appendix Fig Source Data [file 44319_2025_491_MOESM11_ESM.zip › EV/EV2/EV2D/EV2D.png]

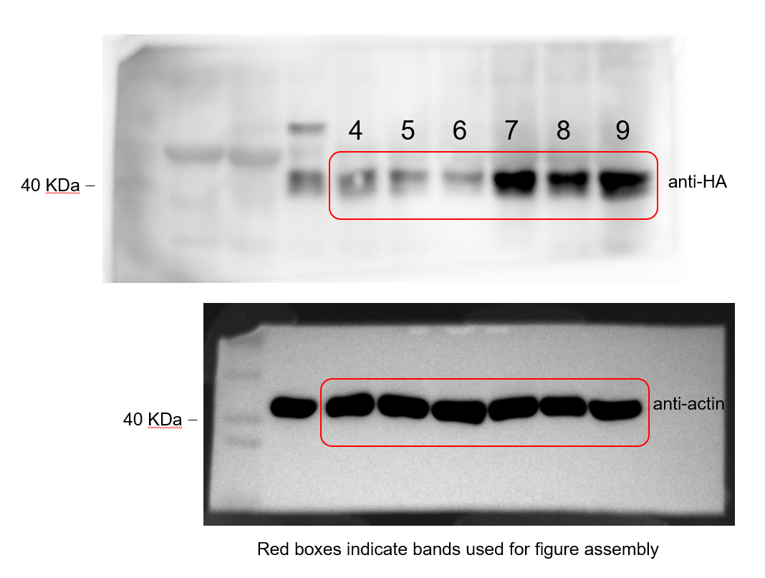

Supplement: Supplementary file 11 — EV+Appendix Fig Source Data [file 44319_2025_491_MOESM11_ESM.zip › EV/EV2/EV2F/EV2F.png]

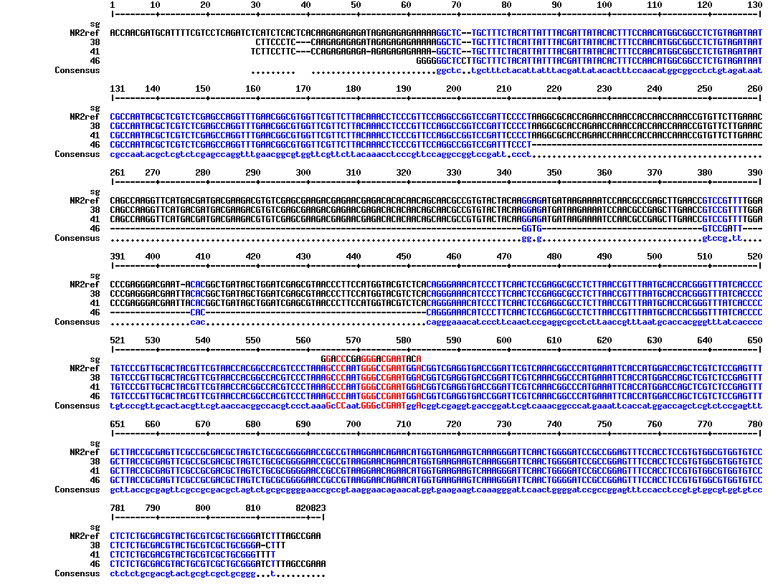

Supplement: Supplementary file 11 — EV+Appendix Fig Source Data [file 44319_2025_491_MOESM11_ESM.zip › EV/EV2/EV2G/EV2G.png]

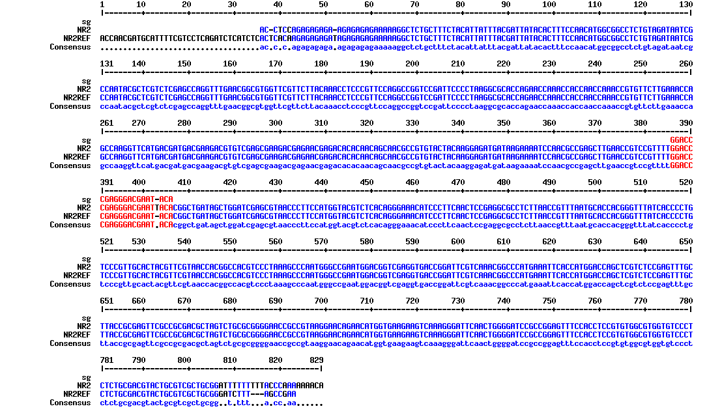

Supplement: Supplementary file 11 — EV+Appendix Fig Source Data [file 44319_2025_491_MOESM11_ESM.zip › EV/EV2/EV2I/EV2I.png]

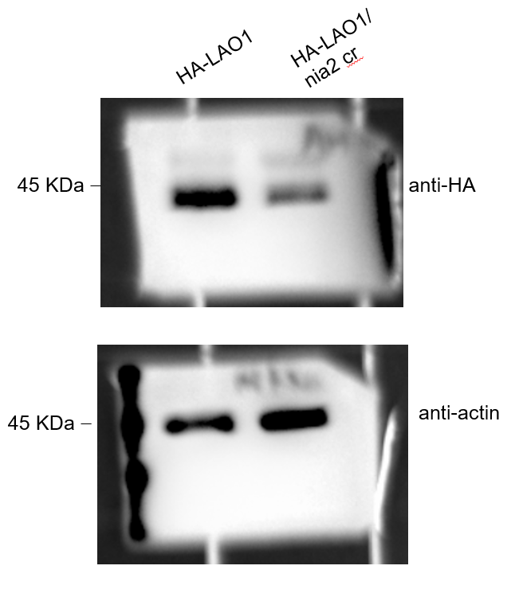

Supplement: Supplementary file 11 — EV+Appendix Fig Source Data [file 44319_2025_491_MOESM11_ESM.zip › EV/EV2/EV2J/EV2J.png]

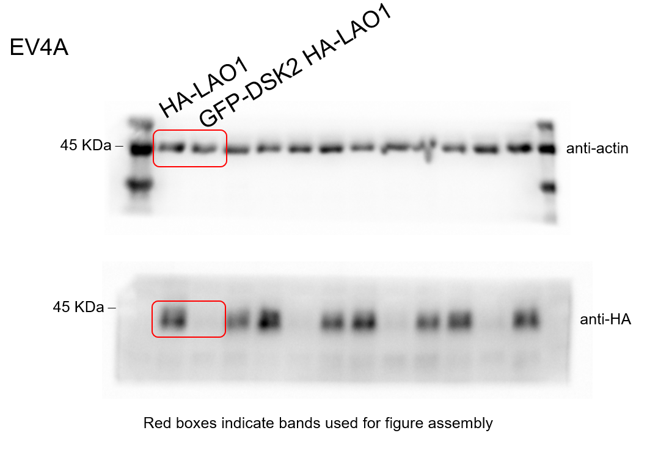

Supplement: Supplementary file 11 — EV+Appendix Fig Source Data [file 44319_2025_491_MOESM11_ESM.zip › EV/EV3/EV3A/EV3A.png]

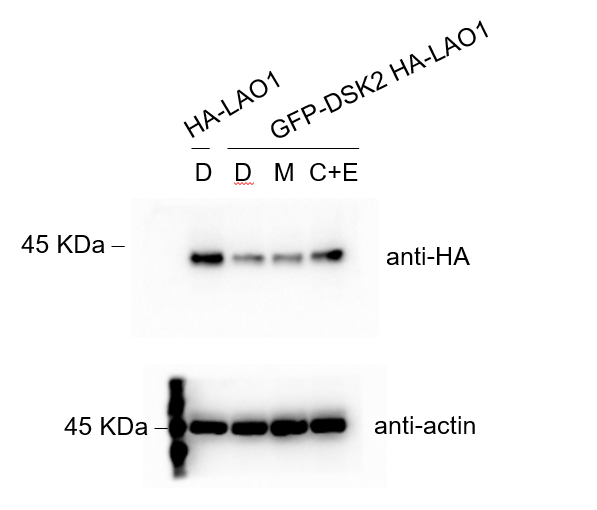

Supplement: Supplementary file 11 — EV+Appendix Fig Source Data [file 44319_2025_491_MOESM11_ESM.zip › EV/EV3/EV3B/EV3B.png]

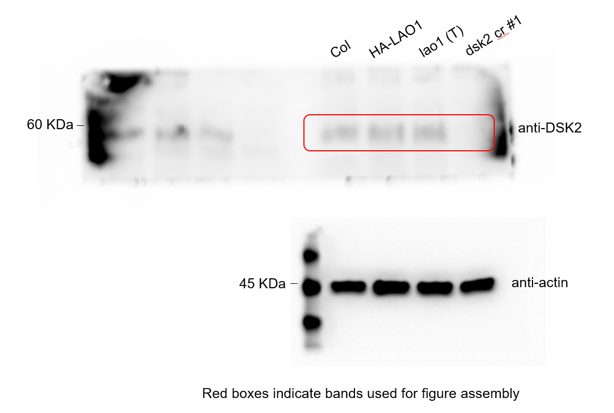

Supplement: Supplementary file 11 — EV+Appendix Fig Source Data [file 44319_2025_491_MOESM11_ESM.zip › EV/EV3/EV3C/EV3C.png]

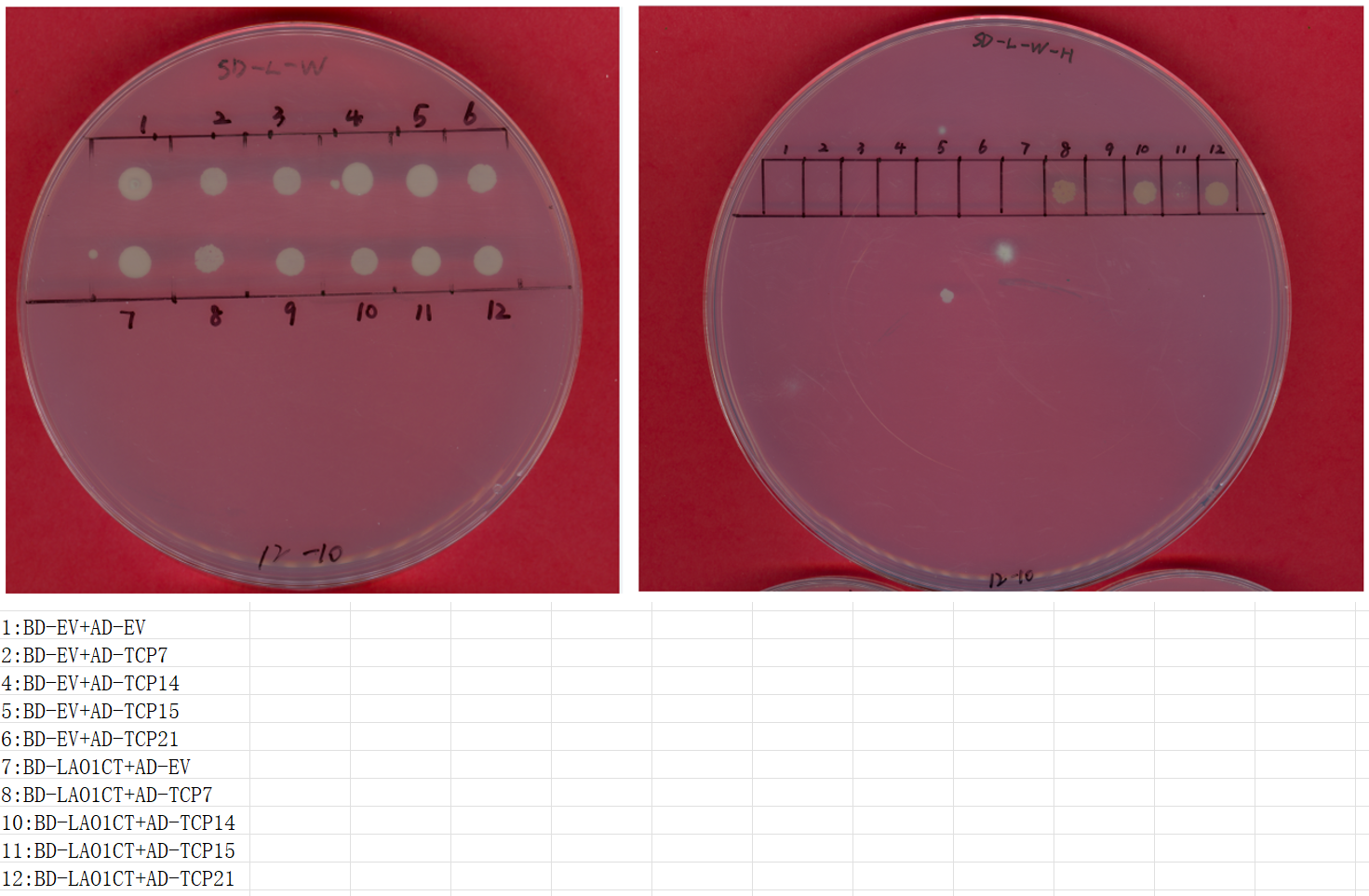

Supplement: Supplementary file 11 — EV+Appendix Fig Source Data [file 44319_2025_491_MOESM11_ESM.zip › EV/EV4/EV4B/EV4B.png]

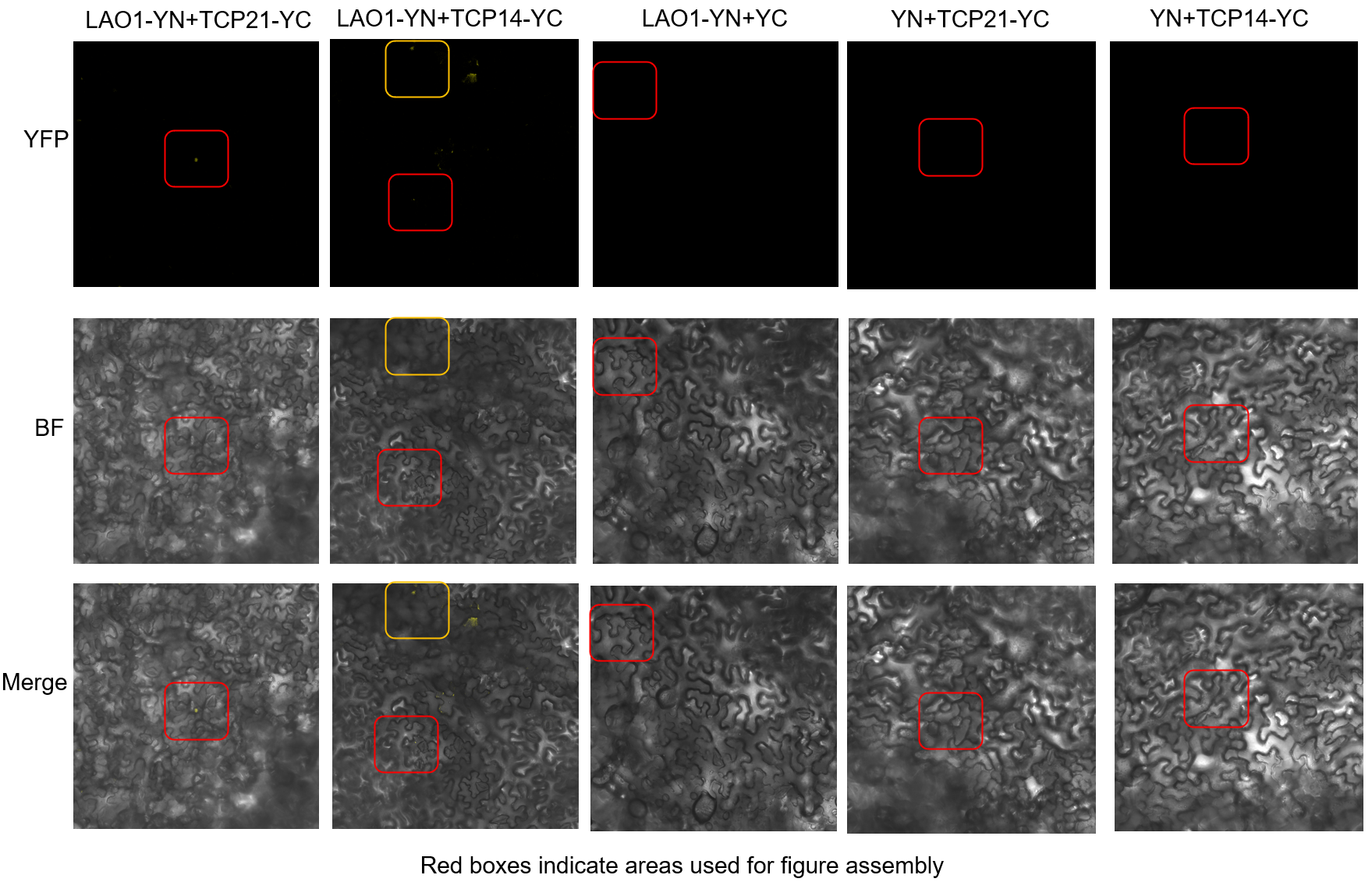

Supplement: Supplementary file 11 — EV+Appendix Fig Source Data [file 44319_2025_491_MOESM11_ESM.zip › EV/EV4/EV4C/EV4C-compiled.png]

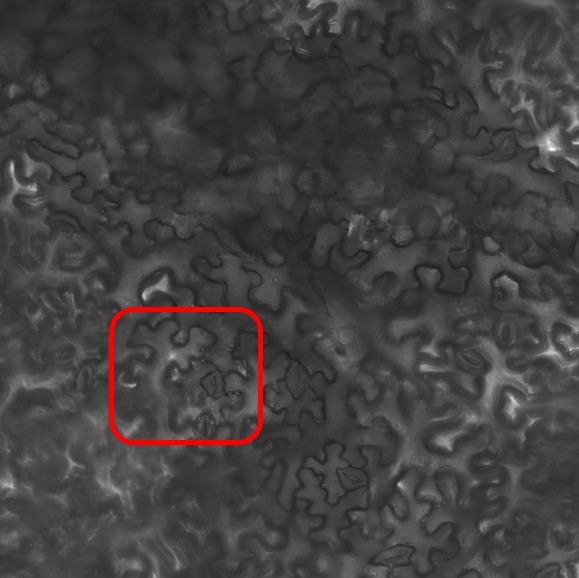

Supplement: Supplementary file 11 — EV+Appendix Fig Source Data [file 44319_2025_491_MOESM11_ESM.zip › EV/EV4/EV4C/LAO1-YN+TCP14-YC (BF).jpg]

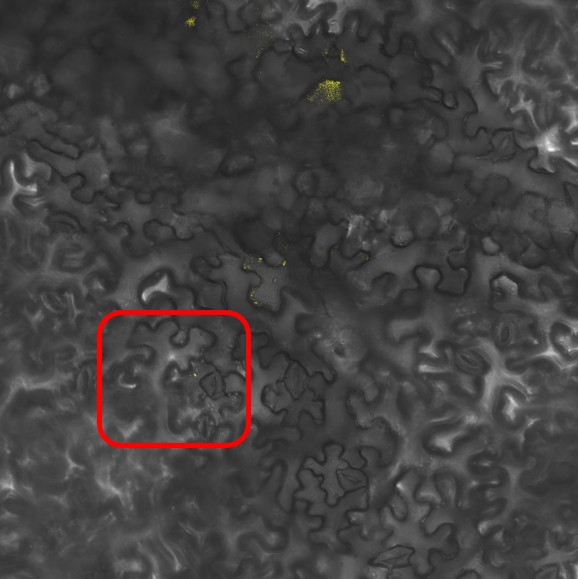

Supplement: Supplementary file 11 — EV+Appendix Fig Source Data [file 44319_2025_491_MOESM11_ESM.zip › EV/EV4/EV4C/LAO1-YN+TCP14-YC (Merge).jpg]

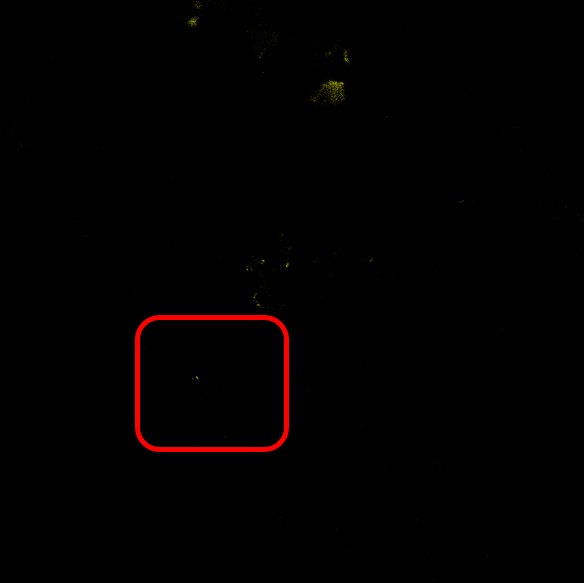

Supplement: Supplementary file 11 — EV+Appendix Fig Source Data [file 44319_2025_491_MOESM11_ESM.zip › EV/EV4/EV4C/LAO1-YN+TCP14-YC (YFP).jpg]

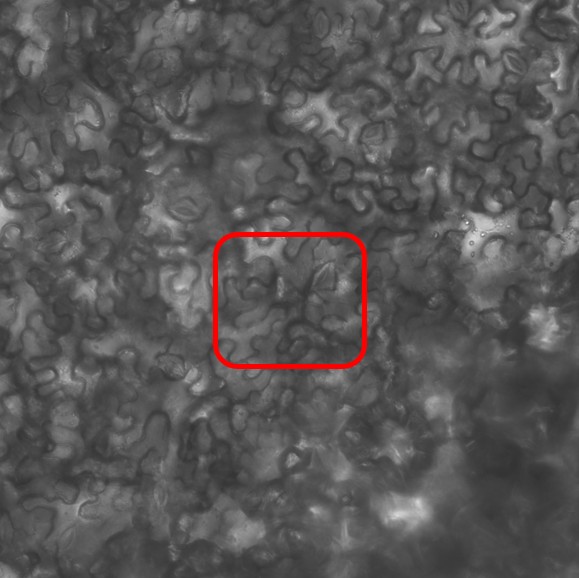

Supplement: Supplementary file 11 — EV+Appendix Fig Source Data [file 44319_2025_491_MOESM11_ESM.zip › EV/EV4/EV4C/LAO1-YN+TCP21-YC (BF).jpg]

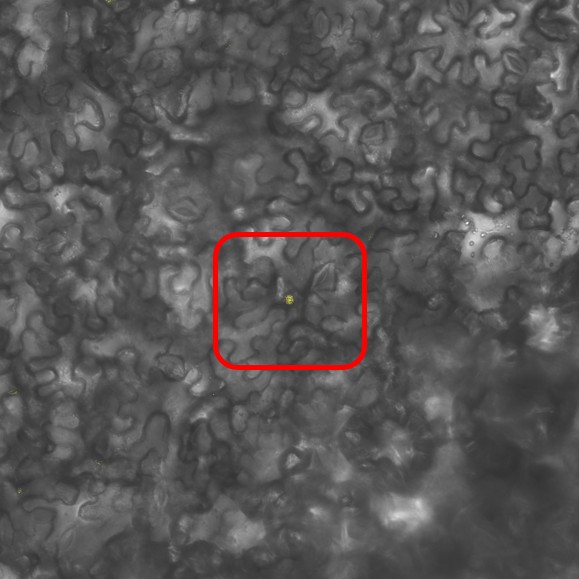

Supplement: Supplementary file 11 — EV+Appendix Fig Source Data [file 44319_2025_491_MOESM11_ESM.zip › EV/EV4/EV4C/LAO1-YN+TCP21-YC (Merge).jpg]

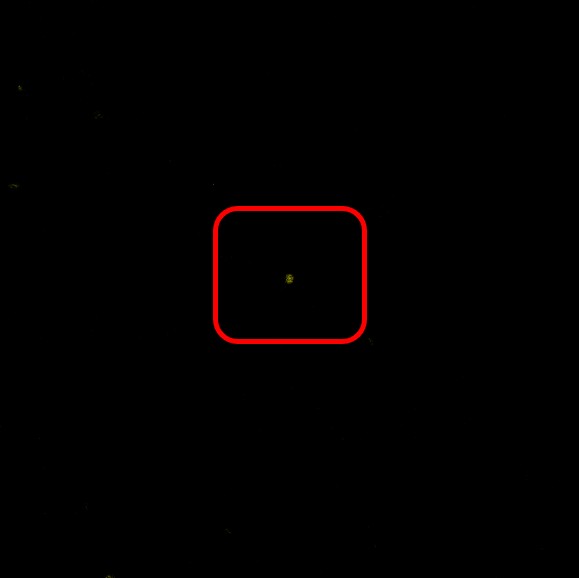

Supplement: Supplementary file 11 — EV+Appendix Fig Source Data [file 44319_2025_491_MOESM11_ESM.zip › EV/EV4/EV4C/LAO1-YN+TCP21-YC (YFP).jpg]

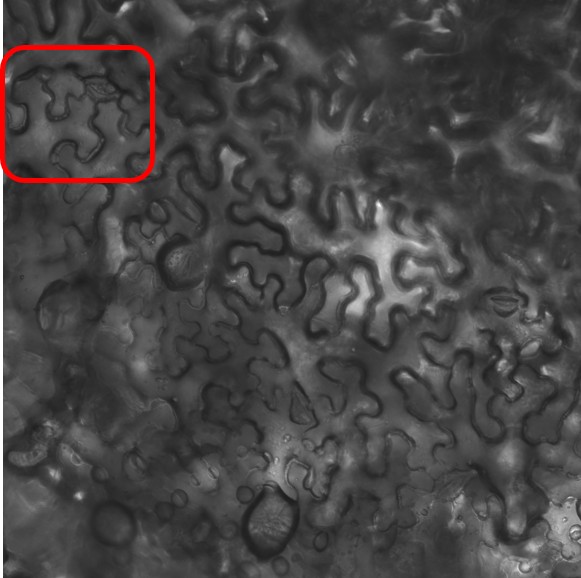

Supplement: Supplementary file 11 — EV+Appendix Fig Source Data [file 44319_2025_491_MOESM11_ESM.zip › EV/EV4/EV4C/LAO1-YN+YC (BF).jpg]

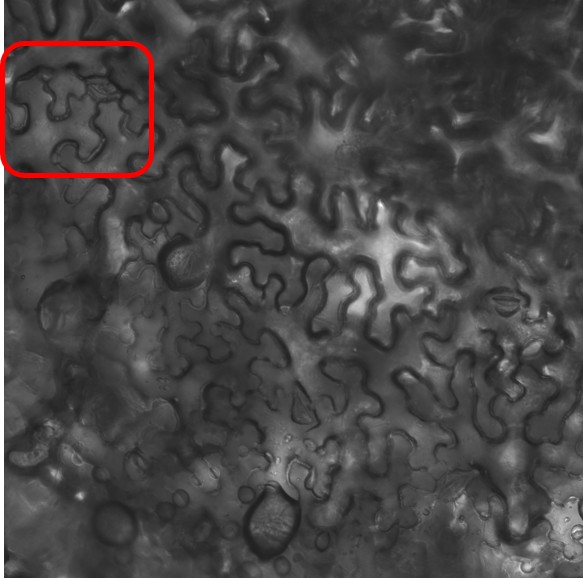

Supplement: Supplementary file 11 — EV+Appendix Fig Source Data [file 44319_2025_491_MOESM11_ESM.zip › EV/EV4/EV4C/LAO1-YN+YC (Merge).jpg]

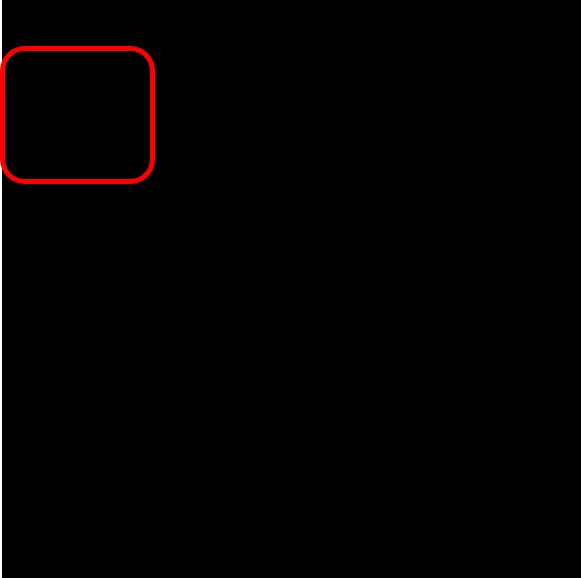

Supplement: Supplementary file 11 — EV+Appendix Fig Source Data [file 44319_2025_491_MOESM11_ESM.zip › EV/EV4/EV4C/LAO1-YN+YC (YFP).jpg]

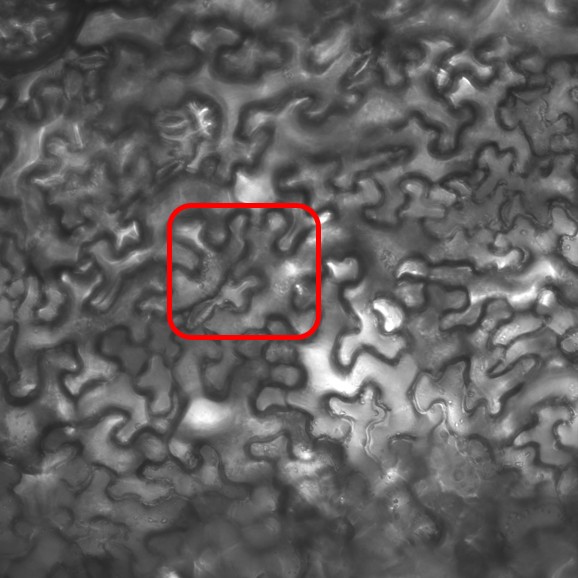

Supplement: Supplementary file 11 — EV+Appendix Fig Source Data [file 44319_2025_491_MOESM11_ESM.zip › EV/EV4/EV4C/YN+TCP14-YC (BF).jpg]

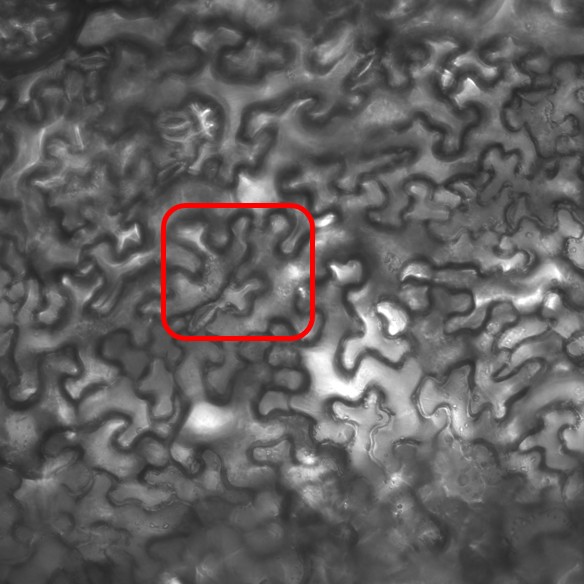

Supplement: Supplementary file 11 — EV+Appendix Fig Source Data [file 44319_2025_491_MOESM11_ESM.zip › EV/EV4/EV4C/YN+TCP14-YC (Merge).jpg]

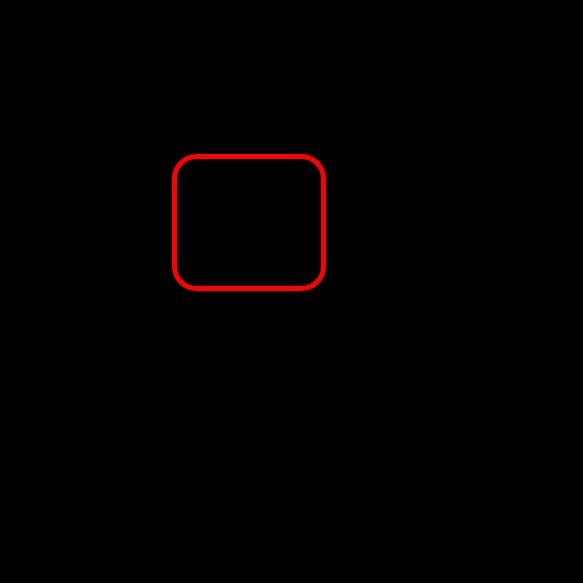

Supplement: Supplementary file 11 — EV+Appendix Fig Source Data [file 44319_2025_491_MOESM11_ESM.zip › EV/EV4/EV4C/YN+TCP14-YC (YFP).jpg]

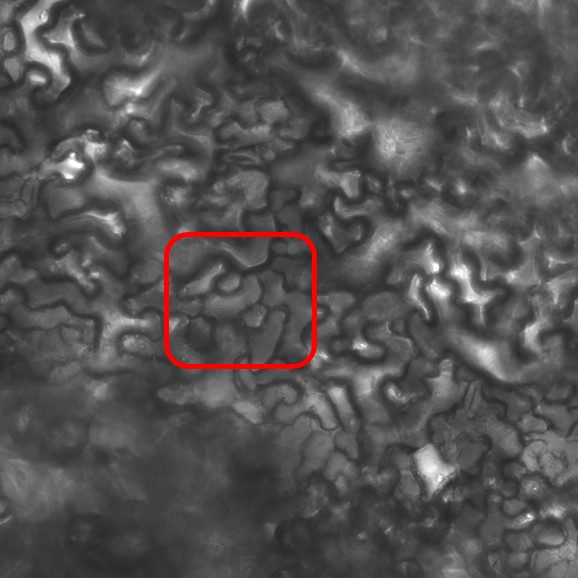

Supplement: Supplementary file 11 — EV+Appendix Fig Source Data [file 44319_2025_491_MOESM11_ESM.zip › EV/EV4/EV4C/YN+TCP21-YC (BF).jpg]

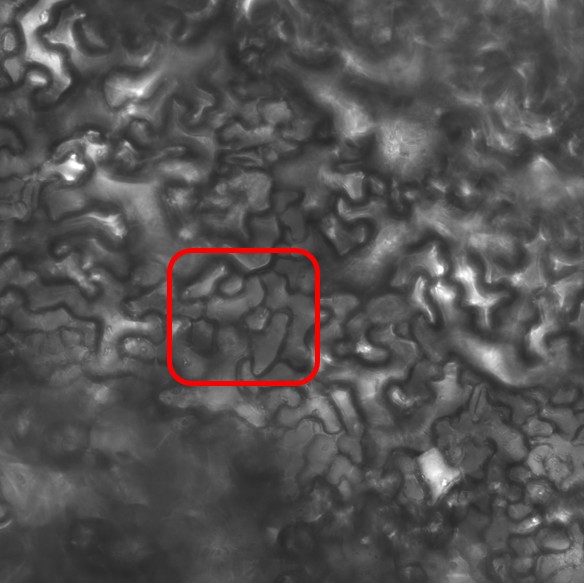

Supplement: Supplementary file 11 — EV+Appendix Fig Source Data [file 44319_2025_491_MOESM11_ESM.zip › EV/EV4/EV4C/YN+TCP21-YC (Merge).jpg]

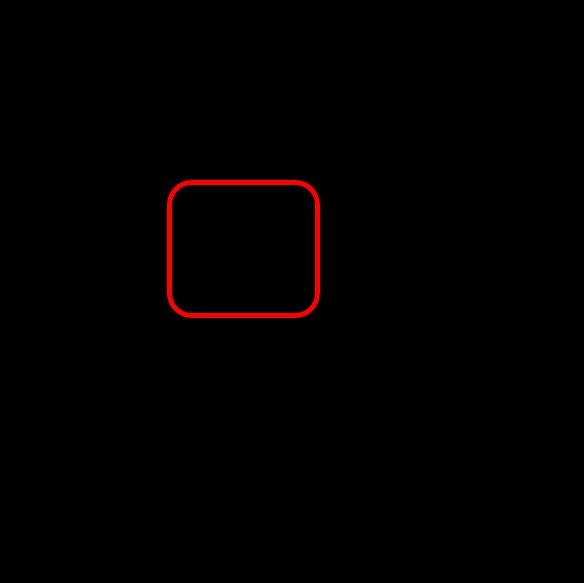

Supplement: Supplementary file 11 — EV+Appendix Fig Source Data [file 44319_2025_491_MOESM11_ESM.zip › EV/EV4/EV4C/YN+TCP21-YC (YFP).jpg]

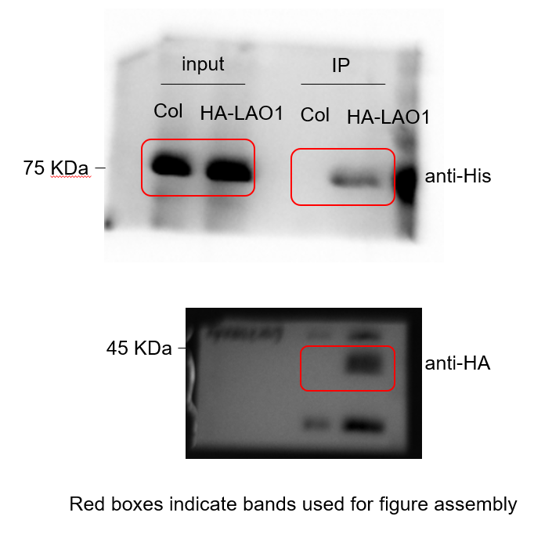

Supplement: Supplementary file 11 — EV+Appendix Fig Source Data [file 44319_2025_491_MOESM11_ESM.zip › EV/EV4/EV4D/EV4D.png]

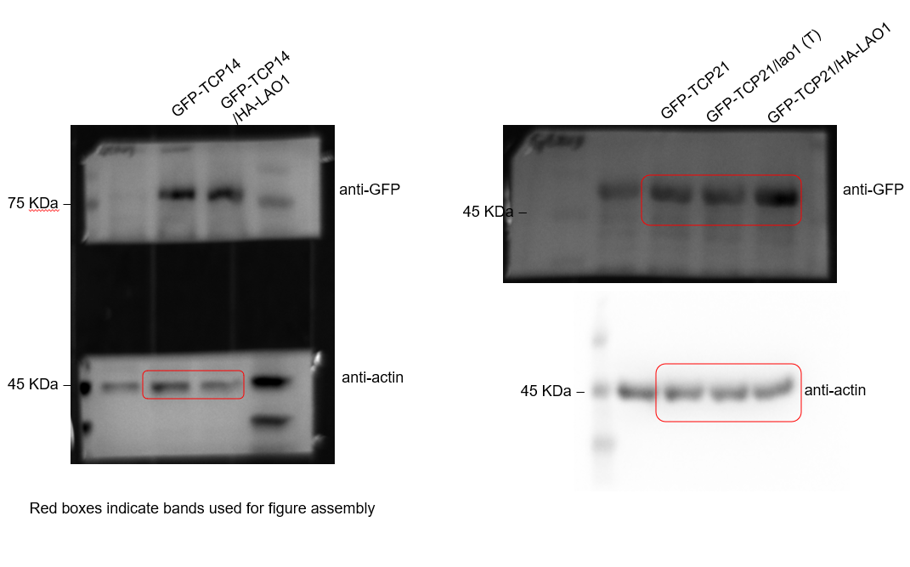

Supplement: Supplementary file 11 — EV+Appendix Fig Source Data [file 44319_2025_491_MOESM11_ESM.zip › EV/EV4/EV4E/EV4E.png]
