# Supplementary material for: DSK2-mediated degradation of F-box protein LAO1 and class I TCPs modulates the nitrogen starvation response
Source: EMBO Rep. 2025 May 30;26(14):3614–39. doi: 10.1038/s44319-025-00491-9 (PMC12287301; doi:10.1038/s44319-025-00491-9)
Supplement: Supplementary file 12 — Expanded View Figures [file 44319_2025_491_MOESM12_ESM.pdf]

Expanded View Figures

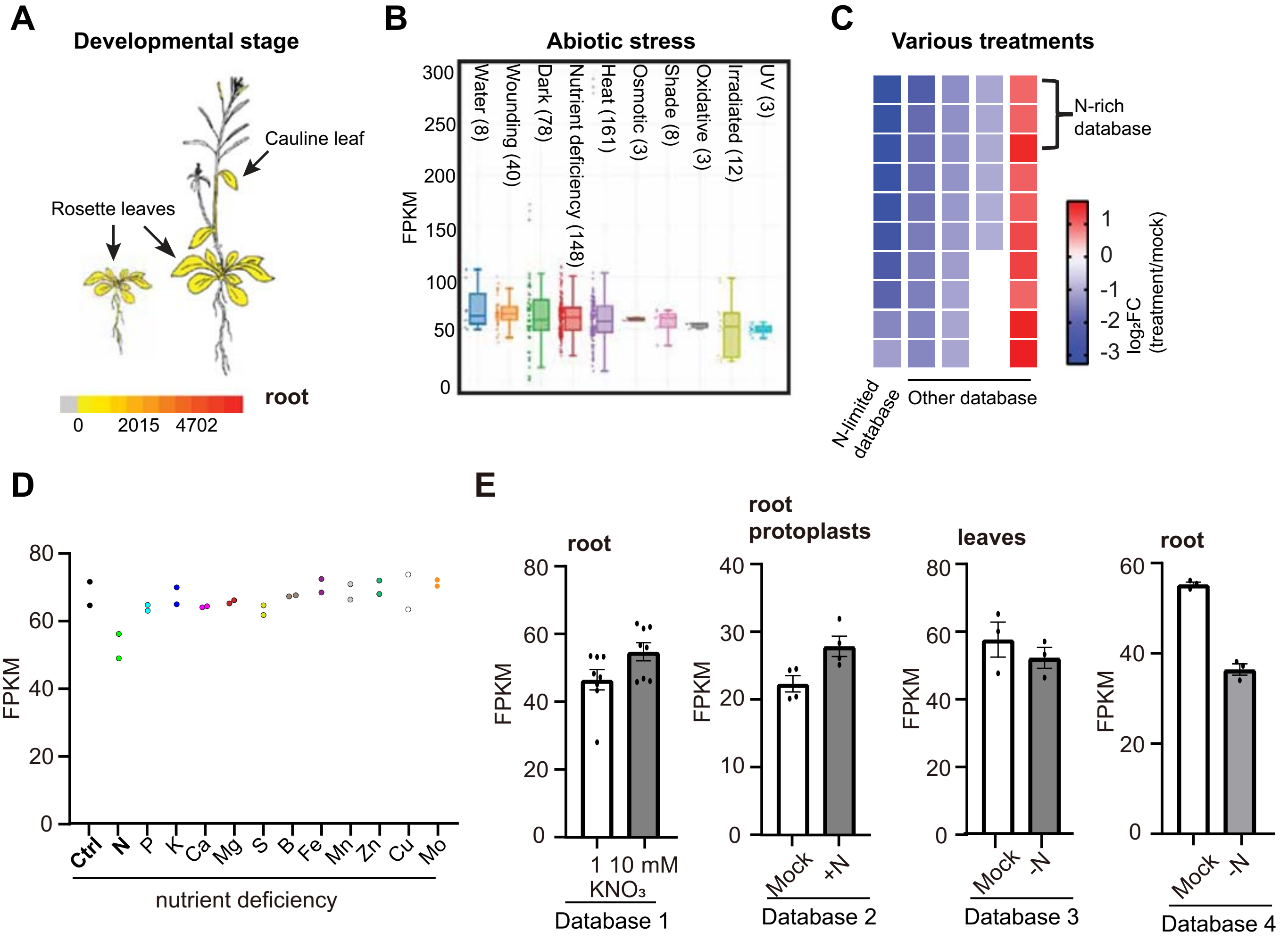

Figure EV1. ***LAO1* is a nitrogen-responsive gene.**

(**A**) *LAO1* predominantly expressed in rosette and cauline leaves. Data were retrieved from *Arabidopsis* eFP browser. (**B**) The expression level of *LAO1* (as determined by the mean FPKM of *LAO1* under the conditions in each category) among top 10 abiotic stress conditions. FPKM, Fragments Per Kilobase of transcript per Million mapped reads. The graph was generated using online tools (https://plantrnadb.com/athrdb/). (**C–E**) *LAO1* expression level was tightly associated with nitrogen availability. (**C**) A heat-map representation of the expression levels of *LAO1* in 46 RNA-seq databases from various treatments. Each square represents the log_2FC (treatment/mock) of *LAO1* in each treatment. (**D**) Deficiency of nitrogen, but not other nutrients, downregulated the expression of *LAO1* gene. Data were shown as individual datapoints of two independent replicates. (**E**) Elevated nitrogen level promoted *LAO1* expression in both root and root protoplasts (Databases 1&2), while nitrogen starvation inhibited *LAO1* expression in both leaves and root (Databases 3&4). Data were shown in mean ± SEM of at least 3 biological replicates. The databases used for gene expression analyses of LAO1 in (**C**), (**D**), and (**E**) were listed in Dataset EV1 file.

 © The Author(s)

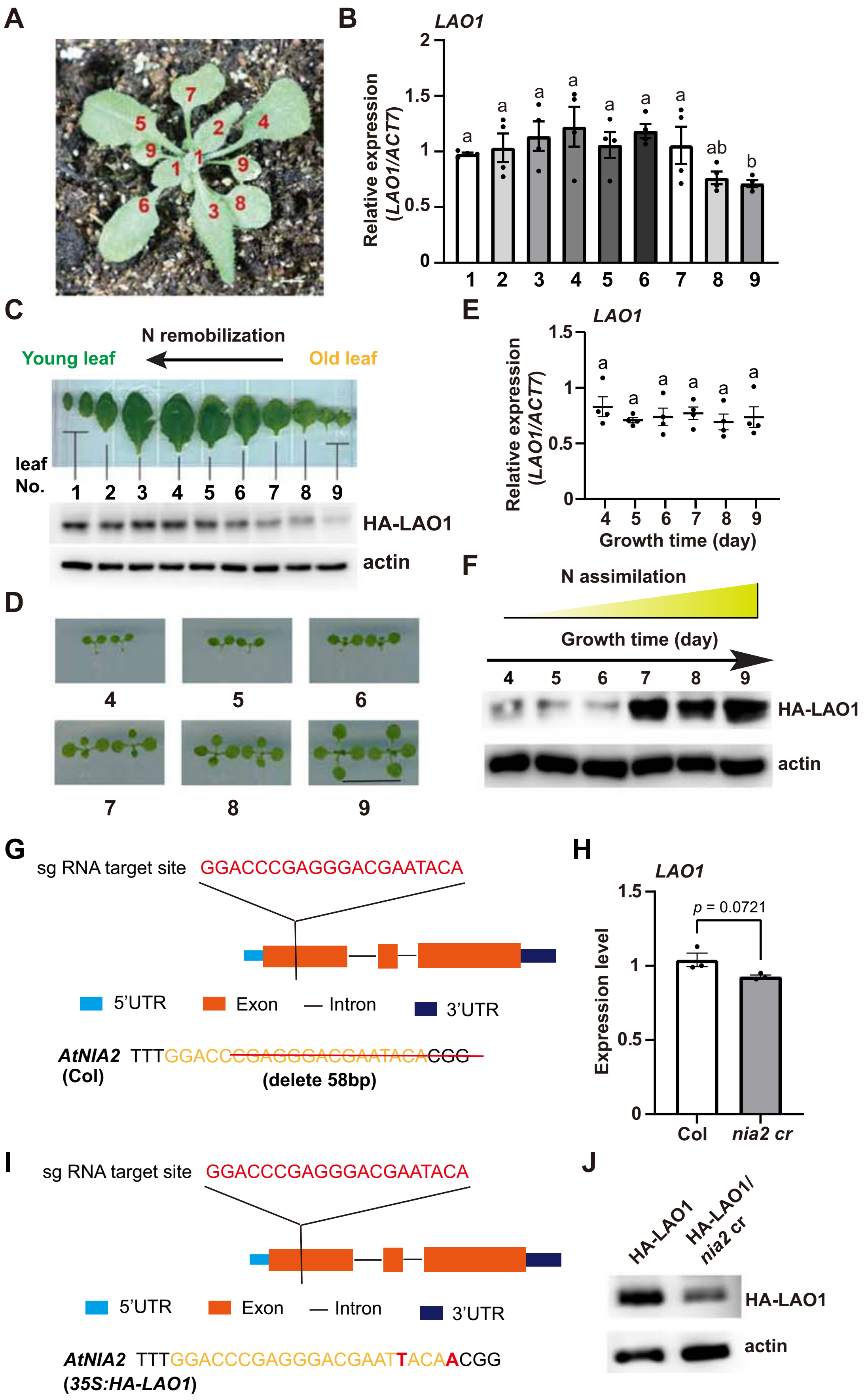
A
B
LAO1
Relative expression (LAO1/ACT7)
Young leaf
N remobilization
Old leaf
leaf No.
HA-LAO1
actin
Growth time (day)
N assimilation
sg RNA target site GGACCCGAGGGACGAATACA
5'UTR
Exon
— Intron
3'UTR
AtNIA2 TTTGGACCCGAGGGACGAATACACGG
(Col)
(delete 58bp)
Expression level
p = 0.0721
Col
nia2 cr
AtNIA2 TTTGGACCCGAGGGACGAATTACAACGG
(35S:HA-LAO1)
HA-LAO1/ nia2 cr

Figure EV2. LAO1 abundance is associated with nitrogen dynamics in plants.

(**A**) A representative graph illustrates the numbering of leaves on an adult plant for downstream applications. The two youngest leaves were grouped together as 1, while the two oldest leaves were grouped together as 9. Scale bar: 1 cm. (**B**) The relative expression levels of *LAO1* in different leaves of 3-week-old Col adult plant. *ACT7* was used as internal control. Data were shown in mean ± SEM of 4 different plants. (**C**) The protein levels of HA-LAO1 in different leaves of 3-week-old *35S: HA-LAO1* adult plant. Total proteins were extracted from similar amounts of leaves with the indicated number, and western blots were performed using anti-HA and anti-actin antibodies. (**D**) A representative graph showing the morphology of seedling grown under continuous white light condition in a growth chamber at 22 °C for 4 to 9 days after stratification (DAS). Scale bar: 1 cm. (**E**) The relative expression levels of *LAO1* in Col seedlings from 4 to 9 DAS. *ACT7* was used as internal control. Data were shown in mean ± SEM of 4 biological replicates. (**F**) The protein levels of HA-LAO1 in *35S: HA-LAO1* seedlings, from 4 to 9 DAS. Total proteins were extracted from *35S: HA-LAO1* seedlings at the indicated growth times. Western blots were performed using anti-HA and anti-actin antibodies. (**G**, **I**) Mutation induced by CRISPR-Cas9 at *NIA2* gene locus. The gene structure of *NIA2* and the position of sgRNA were shown. Mutation was confirmed by PCR-sequencing of targeted locus in both Col (**G**) and *35S: HA-LAO1* transgenic background (**I**). (**H**, **J**) Loss-of-function mutations of NIA2 did not affect *LAO1* mRNA level but downregulated HA-LAO1 protein level. (**H**) The relative expression levels of *LAO1* in 7-day-old Col and *nia2 cr* mutant seedlings. *ACT7* was used as internal control. Data were shown in mean ± SEM of 3 biological replicates. (**J**) Immunoblot analyses of HA-LAO1 protein levels in 7-day-old *35S: HA-LAO1* and *35S: HA-LAO1/nia2 cr* seedlings. Western blot was performed using anti-HA and anti-actin antibodies. In (**B**) and (**E**), statistical significance was determined using one-way ANOVA analysis ($p < 0.05$, ANOVA followed by Tukey's post hoc comparison test). Different letters denoted significant differences. In (**H**), statistical significance was calculated by Student's t test.

 © The Author(s)

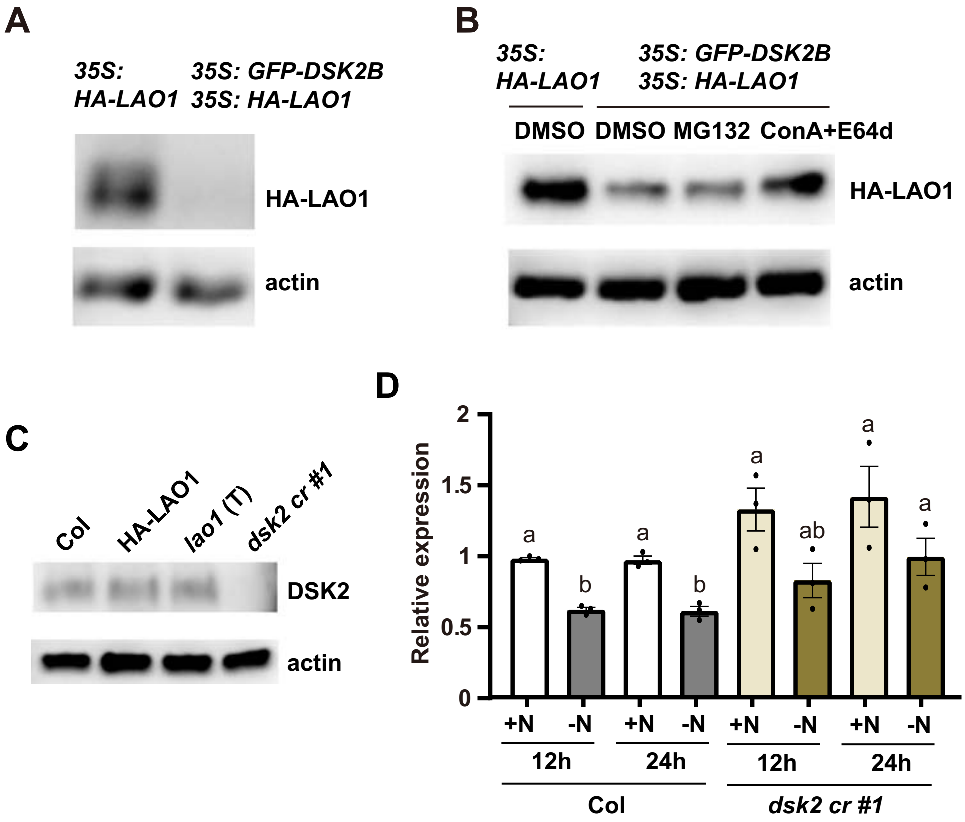

Figure EV3. Analysis of the mutual regulation relationship between DSK2 and LAO1.

(**A**) Overexpression of *DSK2B* reduced HA-LAO1 protein level. Total proteins were extracted from 7-day-old *35S: HA-LAO1* and 35S: *GFP-DSK2B/35S: HA-LAO1* seedlings, and analyzed by immunoblot using anti-HA and anti-actin antibodies. (**B**) ConA and E64d treatment but not MG132 inhibited *DSK2* overexpression induced degradation of HA-LAO1 protein. Seven-day-old *35S: HA-LAO1* and *35S: GFP-DSK2B/35S: HA-LAO1* seedlings were treated with DMSO (control), 50 μM MG132, or 1 μM ConA and 50 μM E64d for 4 h. Total proteins were extracted and analyzed by immunoblot using anti-HA and anti-actin antibodies. (**C**) LAO1 does not affect the protein level of DSK2. Seven-day-old Col*, HA-LAO1*, *lao1*(T), and *dsk2 cr* seedlings were harvested. Total proteins were extracted and analyzed by immunoblot using anti-DSK2 and anti-actin antibodies. (**D**) RT-qPCR analysis of *LAO1* in the *dsk2* cr mutant following nitrogen starvation treatment. *ACT7* was used as internal control. Data were shown in mean ± SEM of 3 biological replicates. Statistical significances were determined using one-way ANOVA analysis ($p < 0.05$, ANOVA followed by Tukey's post hoc comparison test). Different letters denoted significant differences.

© The Author(s)

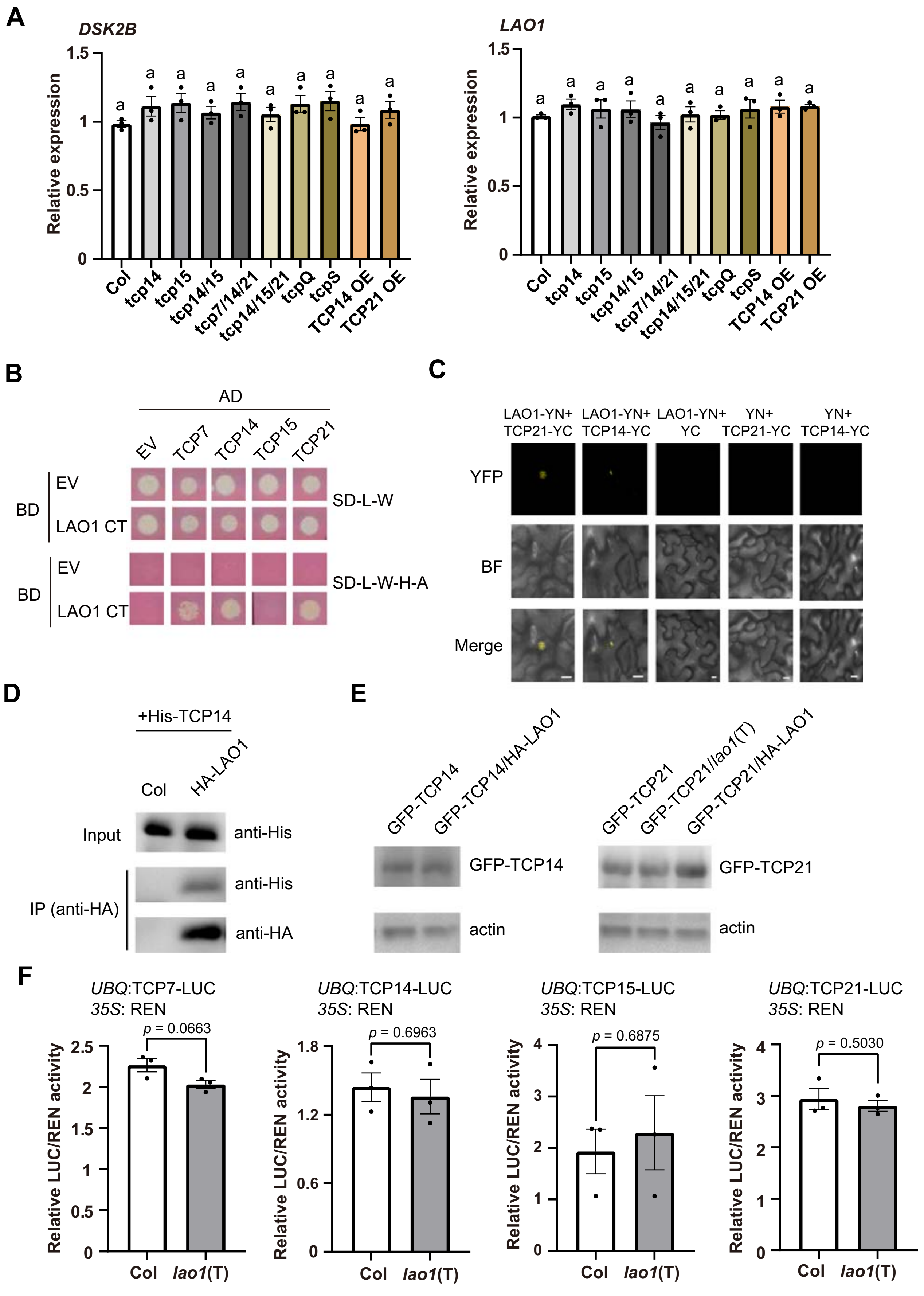
A
DSK2B
LAO1
Relative expression
1.5
1
0.5
0
a
Col
tcp14
tcp15
tcp14/15
tcp7/14/21
tcp14/15/21
tcpQ
tcpS
TCP14 OE
TCP21 OE
B
AD
EV
TCP7
TCP14
TCP15
TCP21
BD
EV
LAO1 CT
SD-L-W
SD-L-W-H-A
C
LAO1-YN+ TCP21-YC
LAO1-YN+ TCP14-YC
LAO1-YN+ YC
YN+ TCP21-YC
YN+ TCP14-YC
YFP
BF
Merge
D
+His-TCP14
Col
HA-LAO1
Input
anti-His
IP (anti-HA)
anti-His
anti-HA
E
GFP-TCP14
GFP-TCP14/HA-LAO1
GFP-TCP21
GFP-TCP21/lao1(T)
GFP-TCP21/HA-LAO1
GFP-TCP14
GFP-TCP21
actin
actin
F
UBQ:TCP7-LUC
35S: REN
p = 0.0663
UBQ:TCP14-LUC
35S: REN
p = 0.6963
UBQ:TCP15-LUC
35S: REN
p = 0.6875
UBQ:TCP21-LUC
35S: REN
p = 0.5030
Relative LUC/REN activity
Col
lao1(T)

© The Author(s)

◀ **Figure EV4. Gene expression analyses of *DSK2* and *LAO1* in *TCP* genetic materials, along with regulatory analyses between LAO1 and TCPs.**

(**A**) TCPs do not affect the expression of *DSK2B* or *LAO1*. *ACT7* was used as internal control. Data were shown in mean ± SEM of 3 biological replicates. The statistical significances were determined using one-way ANOVA analysis ($p < 0.05$, ANOVA followed by Tukey's post hoc comparison test). Different letters denoted significant differences. (**B**) LAO1 interacted with TCPs in yeast. Corresponding constructs were co-transformed into the yeast strain Y2H-gold. The colonies appeared on SD-L-W plates were transferred onto SD-L-W-H and SD-L-W-A-H plates for interaction test. (**C**) BiFC assay in leaves of one-month-old *N.benthamiana* plants using Agrobacteria GV3101 mediated transient expression. Scale bars: 20 µm. (**D**) HA-LAO1 interacted with recombinant His-TCP14 in semi-in vivo pull-down assays. HA-LAO1 proteins were immunoprecipitated from transgenic *35S: HA-LAO1* plants, and then incubated with recombinant His-TCP14 protein. The pellets were boiled in 2×SDS loading buffer, and the samples were detected by western blotting using anti-His and anti-HA antibodies. (**E**) Immunoblot analyses of GFP-TCP14 or GFP-TCP21 protein abundance. Total proteins were extracted from the leaves of 7-day-old *GFP-TCP14*, *GFP-TCP14/HA-LAO1*, *GFP-TCP21*, *GFP-TCP21/HA-LAO1*, and *GFP-TCP14/lao1*(T) plants. Western blots were performed using anti-GFP and anti-actin antibodies. (**F**) The luminescence ratio of TCP-LUC/REN in Col and *lao1*(T) mutant protoplasts. Experimental design was similar as Fig. 6E. Relative TCP-LUC/REN activity was shown in mean ± SEM of 3 independent experiments. All the statistical significances were calculated by the Student's *t* test.

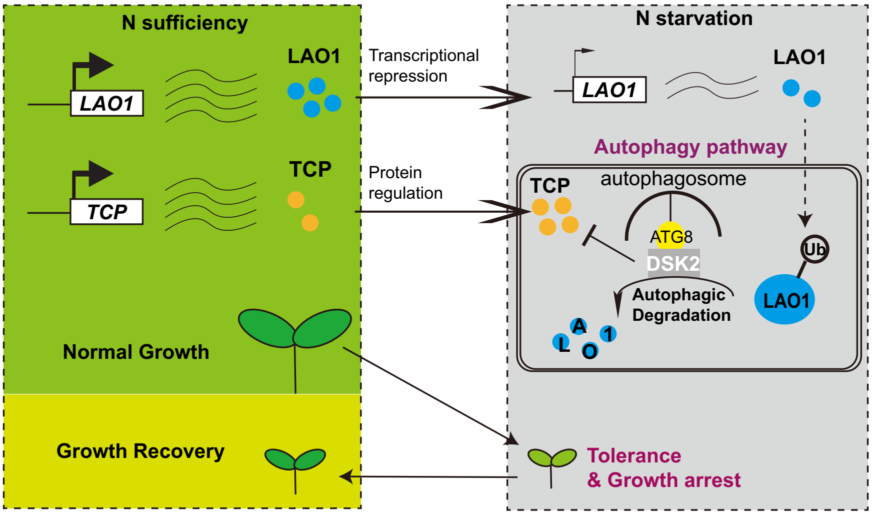

Figure EV5. A simplified model for deciphering the role of DSK2-LAO1-TCP module in plant response to nitrogen starvation.

Under N-sufficient conditions, active N assimilation maintains high levels of LAO1, while relatively lower levels of TCP proteins, supporting optimal growth. Upon N starvation, LAO1 protein is degraded through DSK2-mediated autophagy, whereas TCP proteins accumulate, enhancing plant tolerance to nitrogen starvation. Concurrently, DSK2 modulates TCP protein stability to balance growth and tolerance.

 © The Author(s)
